# Dose-responses for mortality from cerebrovascular and heart diseases in atomic bomb survivors: 1950–2003

**DOI:** 10.1007/s00411-017-0722-5

**Published:** 2017-12-08

**Authors:** Helmut Schöllnberger, Markus Eidemüller, Harry M. Cullings, Cristoforo Simonetto, Frauke Neff, Jan Christian Kaiser

**Affiliations:** 1 Department of Radiation Sciences, Institute of Radiation Protection, Helmholtz Zentrum München, Ingolstädter Landstrasse 1, 85764 Neuherberg, Germany; 2 Department of Radiation Protection and the Environment, Federal Office for Radiation Protection, Ingolstädter Landstrasse 1, 85764 Neuherberg, Germany; 3 Department of Statistics, Radiation Effects Research Foundation, Hiroshima, Japan; 4 Institute of Pathology, Städtisches Klinikum München and Technical University of Munich, Munich, Germany

**Keywords:** Ionizing radiation, Cardiovascular diseases, Atomic bomb survivors, Risk assessment, Linear no-threshold model, Nonlinear dose–response

## Abstract

The scientific community faces important discussions on the validity of the linear no-threshold (LNT) model for radiation-associated cardiovascular diseases at low and moderate doses. In the present study, mortalities from cerebrovascular diseases (CeVD) and heart diseases from the latest data on atomic bomb survivors were analyzed. The analysis was performed with several radio-biologically motivated linear and nonlinear dose–response models. For each detrimental health outcome one set of models was identified that all fitted the data about equally well. This set was used for multi-model inference (MMI), a statistical method of superposing different models to allow risk estimates to be based on several plausible dose–response models rather than just relying on a single model of choice. MMI provides a more accurate determination of the dose response and a more comprehensive characterization of uncertainties. It was found that for CeVD, the dose–response curve from MMI is located below the linear no-threshold model at low and medium doses (0–1.4 Gy). At higher doses MMI predicts a higher risk compared to the LNT model. A sublinear dose–response was also found for heart diseases (0–3 Gy). The analyses provide no conclusive answer to the question whether there is a radiation risk below 0.75 Gy for CeVD and 2.6 Gy for heart diseases. MMI suggests that the dose–response curves for CeVD and heart diseases in the Lifespan Study are sublinear at low and moderate doses. This has relevance for radiotherapy treatment planning and for international radiation protection practices in general.

**Electronic supplementary material:**

The online version of this article (10.1007/s00411-017-0722-5) contains supplementary material, which is available to authorized users.

## Introduction

High doses of ionizing radiation (IR) can cause non-cancer diseases including cardiovascular-related detrimental health outcomes. Such evidence stems from analyzing cohorts of radiotherapy patients (Darby et al. 2013) and animal experiments (Stewart et al. 2006). One cohort that is very important for IR-associated risk analyses and radiation protection is the Life Span Study (LSS). It includes information on high-, medium- and low-dose exposed atomic bomb survivors. For cardiovascular diseases (CVD) there are a range of findings including detrimental effects (Preston et al. 2003; Shimizu et al. 2010; Little et al. 2012; Azizova et al. 2012, 2014, 2015a, b; Moseeva et al. 2014; Ozasa et al. 2012, 2017; Gillies et al. 2017), indications for threshold-doses from analyzing LSS data (Schöllnberger et al. 2012) and experimental evidence for anti-inflammatory effects (Mitchel et al. 2007, 2011, 2013; Rödel et al. 2012a, b; Frey et al. 2015; Mathias et al. 2015; Le Gallic et al. 2015).

With respect to cancer the linear no-threshold (LNT) model is applied in international radiation protection practices. This has been challenged and the discussions continue for CVD. As for cancer, these debates relate to the questions which dose–response models should be applied to radio-epidemiological cohorts, whether they should include threshold and other nonlinear models and whether risk increases linearly from lowest doses (such as those occurring due to environmental exposures) up to the high doses applied in radiotherapy (Little et al. 2012, 2013; Schöllnberger and Kaiser 2012; Schöllnberger et al. 2013). Ongoing discussions reflect the controversial nature of this issue (Little 2016). The question of IR-induced risks for CVD is of great importance to the societies given the widespread and increasing use of medical applications such as CT scans and radiotherapy as well as in the context of nuclear energy production and accident related long-term risks.

In the present study, the latest publically available LSS mortality data for two important detrimental health outcomes (cerebrovascular diseases (CeVD) and heart diseases) were analyzed with several radio-biologically motivated nonlinear dose–response models in addition to the LNT model. Multi-model inference (MMI) (Burnham and Anderson 2002; Claeskens and Hjort 2008) techniques were applied to obtain more realistic risk predictions. This stochastic technique has been introduced to radiation epidemiology by Walsh and Kaiser (2011) who also discuss MMI applications in other fields of research. It has also been used in subsequent radio-epidemiological studies (Kaiser et al. 2012; Schöllnberger et al. 2012; Kaiser and Walsh 2013; Simonetto et al. 2015). The present study extends the analysis of Shimizu et al. (2010) in an important manner because MMI provides both a more accurate determination of the dose response and a more comprehensive characterization of model uncertainties by accounting for a possible bias from model selection. Our MMI approach aims to detect nonlinearities in the dose response by extensive analysis applying biologically plausible dose–response models. For CVD it has been applied to the Mayak workers cohort (Simonetto et al. 2015) and an earlier LSS data set (Schöllnberger et al. 2012). Our approach can be considered complementary to the meta-analysis of Little et al. (2012) and Little (2016) who relied on studies with LNT as a foregone conclusion.

## Materials and methods

Two detrimental health outcomes of the latest publically available data of the atomic bomb survivors were analyzed with a larger set of linear and nonlinear dose–response models with the aim of improved understanding of potential risks (or benefits) at low and moderate doses of ionizing radiation.

### Study population

The LSS cohort consists of 86,611 atomic bomb survivors whose individual radiation doses were estimated using the DS02 dosimetry system, considering the location and shielding of each survivor at the time of the nuclear explosions. It includes a large proportion of the survivors who were within 2.5 km of the hypocentres at the time of the bombings and still resided in Hiroshima or Nagasaki in 1950, plus a random age- and sex-matched sample of people 2.5–10 km from the hypocentres who sustained small to negligible radiation doses. This study population was of all ages and both sexes at the time of the bombings (Shimizu et al. 2010). As in the primary analysis (Shimizu et al. 2010), all analyses of the present study were performed using weighted colon doses in Gy, i.e. sum of the γ-ray dose estimate plus 10 times the neutron dose estimate. The person-year weighted mean dose within the whole cohort is 0.116 Gy and the person-year weighted mean dose of the mortality cases is 0.111 Gy. The doses range from 0 to 4 Gy (Shimizu et al. 2010; Preston et al. 2003). As it is visible from Table S1 of the Online Resource, the cohort covers a wide range of doses but is weighted towards low doses, which indicates that it has considerable capability to examine risks at low doses and to examine the shape of the dose–response curve (Shimizu et al. 2010).

The follow-up of the vital status took place from October 1 1950 until the end of 2003. As in the study by Shimizu et al. (2010), the data set with the full follow-up period (1950–2003) was analyzed including distal and proximal survivors (who were within a radius of 3 km from the hypocenters at the time of bombings). This amounted to 86,611 individuals (35,687 men and 50,924 women). During the follow-up period 9622 persons died from CeVD and 8463 died from heart diseases. The number of person-years contained in the data is 3,294,282 (1,280,797 and 2,013,485 for males and females, respectively). Data pertaining to men and women were fitted jointly.

The data are categorized by city, sex, age at exposure, attained age, calendar time and DS02 weighted colon dose. They are provided within a detailed summary table that contains for each available combination of categories the number of subjects, the number of person-years at risk, person-year weighted mean age at exposure, person-year weighted mean attained age, person-year weighted colon dose and the number of cases of death for the different detrimental health outcomes. In the present study, the mortality data that have CeVD and CVD excluding CeVD (generally referred to as heart diseases) as the underlying cause of death were analyzed, corresponding to the main analysis from Shimizu et al. (2010). CeVD are defined by ICD-9 codes 430–438 and comprise hemorrhagic and ischemic diseases (CeVD are often referred to as stroke). CVD excluding CeVD are defined by ICD-9 codes 393–429 (excluding 401, 403, and 405). They contain chronic rheumatic heart diseases, hypertensive and ischemic heart diseases, diseases of pulmonary circulation and other forms of heart diseases. The data-set represents the latest publically available grouped LSS data (Shimizu et al. 2010; data file lsscvd10.dat available at http://www.rerf.jp/library/dl_e/lsscvd10.html). For a more detailed data classification the reader is referred to the Online Resource, Table S1.

In the primary analysis (Shimizu et al. 2010), the data had been analyzed using a stratified baseline model (in opposition to our approach of a parametric baseline, a stratified baseline contains one free parameter for each combination of available categories in the data) combined with LNT, Q and linear-threshold (LTH) models as excess relative risk (ERR) models.

### Risk models

**Fig. 1:**
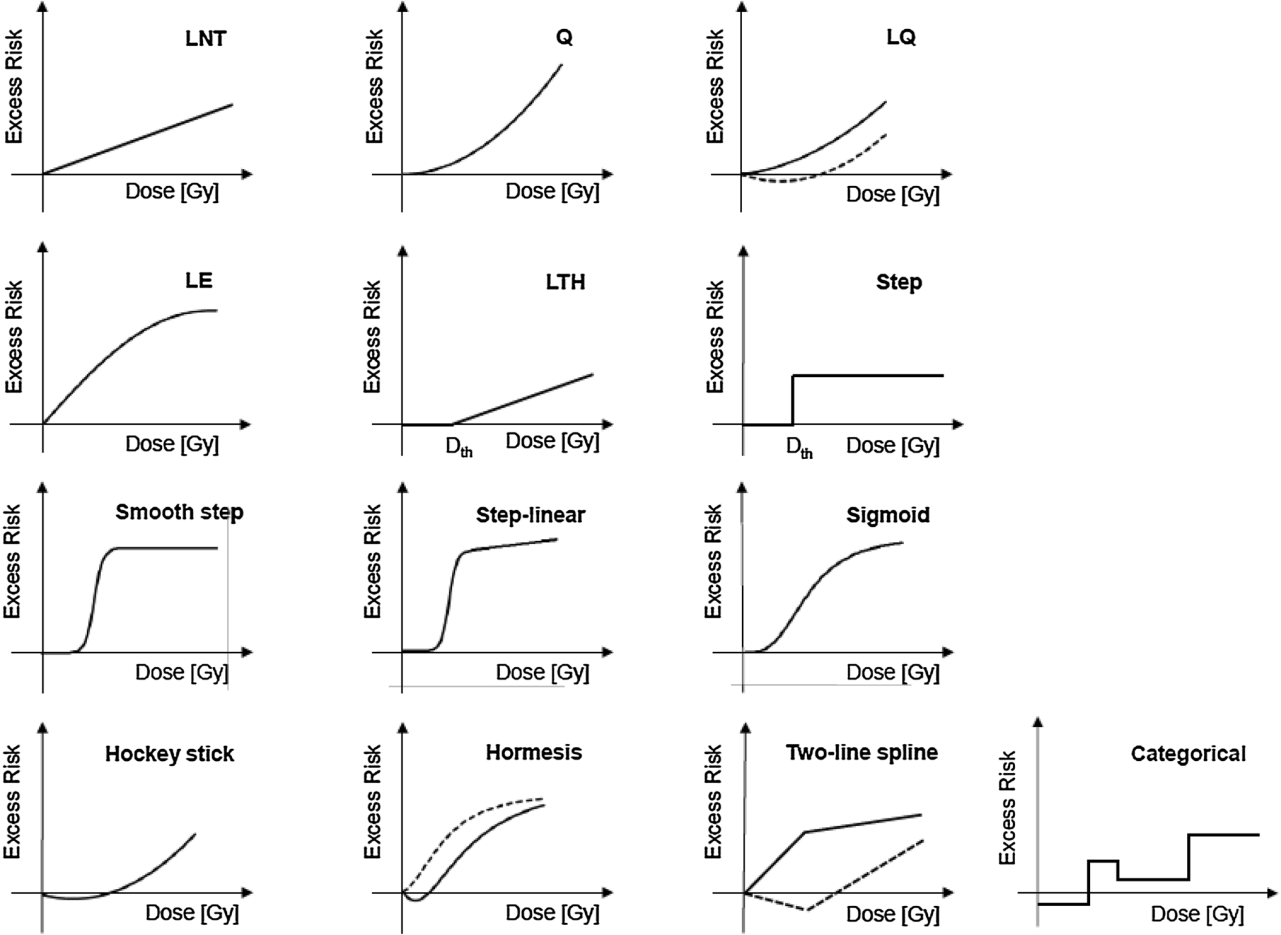
Typical shapes of the functions that were used to analyze the dose responses. 1st row: LNT model, quadratic (Q), linear-quadratic (LQ); 2nd row: linear-exponential (LE), linear-threshold (LTH), step-model; 3rd row: smooth step, step-linear, sigmoid; 4th row: hockey stick (J-shaped model), hormesis, two-line spline, categorical model. Additional dashed lines show the flexibility of some of the models. The mathematical equations for all of these models are provided on pages 6–8 of the Online Resource

The data were analyzed with parametric baseline models that were combined with 13 different dose–response models as ERR model and excess absolute risk (EAR) model. The general form of an ERR model is *h* = *h*_0_ × (1 + *ERR*(*D, s, a, e*)) where *h* is the total hazard function, *h*_0_ is the baseline model and *ERR*(*D, s, a, e*) describes the change of the hazard function with weighted colon dose *D* allowing for dose-modification by sex (*s*), attained age (*a*) and age at exposure (*e*). It is *ERR*(*D, s, a, e*) = *err*(*D*) × ε(*s, a, e*). Here, *err*(*D*) represents one of the dose–response models from Fig. 1 and ε(*s, a, e*) contains the dose–effect modifiers (DEMs) sex, attained age, and age at exposure. The general form of an EAR model is *h* = *h*_0_ + *EAR*(*D, s, a, e*) with *EAR*(*D, s, a, e*) =* ear*(*D*) × ε(*s, a, e*). Here, *ear*(*D*) represents one of the models from Fig. 1. For the parametric baseline model the same functional form has been applied that had been introduced by Preston et al. (2003) and applied by Schöllnberger et al. (2012). For mathematical details see Online Resource, Eq. (S1) and page 6. The models from Fig. 1 are motivated by various results from radiobiological studies including findings that indicate protective effects (i.e. U-shaped or J-shaped dose–responses; Mitchel et al. 2011, 2013; Le Gallic et al. 2015; Mathias et al. 2015) as well as supra-linear responses indicating that the radiation may have acted stronger than predicted by the LNT model. Models such as the LTH and two-line spline models are frequently used within radio-epidemiological studies (e.g. Preston et al. 2003; Shimizu et al. 2010; Ozasa et al. 2012; Kaiser and Walsh 2013, Hsu et al. 2013; Takahashi et al. 2017; Grant et al. 2017). The dose–response models were chosen to reflect many biologically plausible shapes for dose–responses which are deemed adequate to describe the dose response. Generally, both supra-linear and sub-linear models are considered. Naturally, this selection is subjective but it is motivated in part by possible underlying radio-biological mechanism. This closer link to radiobiology cannot be provided by a purely mathematical exploration of the dose responses using penalized B-splines (Eilers and Marx 1996) or fractional polynomials (Faes et al. 2007).

The threshold-dose parameter (*D*_*th*_) contained in some models (LTH, step, smooth step, step-linear, sigmoid, hockey stick, hormesis, two-line spline) was allowed to be free during the model fits, i.e. *D*_*th*_ was estimated during the model fits by allowing it to be optimized. The step model (Fig. 1) with its steep slope usually provides a good estimate of a possible threshold-dose that was then analyzed further with other models (Simonetto et al. 2014). The three step models (step, smooth step and step-linear) were implemented as modified hyperbolic tangent functions, which can accommodate various different shapes. With this function, a step is not imposed a priori but results from fitting that model to data. The mathematical equations for all 13 dose–response models are provided on pages 6–8 of the Online Resource.

### Multi-model inference

MMI is a statistical method of mathematically superposing different non-nested models that all describe a certain data set about equally well (Burnham and Anderson 2002; Claeskens and Hjort 2008). The term MMI has been coined by Burnham and Anderson (2002) for the frequentist approach of model averaging and is consistently used for its application in radiobiology. In contrast to Bayesian model averaging (BMA) (Hoeting et al. 1999), which is based on the evaluation of model-specific marginal likelihood functions to determine a model average, MMI relies on AIC-based model weights. BMA is computationally more demanding and has not yet been applied in radio-epidemiology. We chose MMI over BMA in the present study to be consistent with our previous work (Schöllnberger et al. 2012) to avoid a dependence of our results on the applied methodology.

Both BMA and MMI apply the concept of Occam’s group (Madigan and Raftery 1994; Hoeting et al. 1999; Noble et al. 2009; Kaiser and Walsh 2013) where a group of models deemed adequate for averaging is selected from a much larger group of candidate models (see Fig. 1). The method of picking models for Occam’s group can vary. For example, Walsh and Kaiser (2011) selected all published models, which had been applied to the same LSS data set for the same endpoint (all types of leukaemia), whereas Kaiser and Walsh (2013) developed a rigorous selection process based on likelihood ratio tests (LRTs), which is also applied in the present study.

### Model selection

At first, the full Preston baseline model was combined with the LNT model and fitted as an ERR-LNT model to the data for CeVD, yielding a final deviance (dev) of 13,409.43. This model contains 30 parameters (29 baseline parameters plus the slope of the LNT model). Subsequently, all baseline parameters were tested for their significance using the LRT (a model is considered an improvement over a nested model with a 95% probability, if the deviance is lowered by at least 3.84 points after adding of one parameter): each baseline parameter was set to 0 and all other model parameters were refitted. Rigorous testing led to a new set of statistically significant baseline parameters with 14 parameters less than the full Preston baseline model (see Online Resource, Table S3). This streamlined baseline model was combined with the dose–response models from Fig. 1 as ERR models and fitted to the data. The same selection protocol involving the LRT was then applied to compare nested dose–response models with each other and to eliminate those nested models with inferior deviance values (see pages 11 and 12 of the Online Resource together with Figure S1 and Table S2 for details). For each surviving dose–response model, the three DEMs (sex, attained age and age at exposure) were tested for statistical significance. Subsequently, the full Preston baseline model was combined with an LNT model and implemented as EAR-LNT model yielding dev = 13407.44. The streamlining process led to a baseline model with 12 parameters less than the full Preston baseline model. This streamlined baseline model was combined with the models from Fig. 1 as EAR models and fitted to the data (see page 12 of the Online Resource for details). This selection protocol leads to a set of final non-nested models that have been used for MMI. This group of models is generally referred to as Occcam’s group (Madigan and Raftery 1994; Hoeting et al. 1999; Kaiser and Walsh 2013). Based on the rather rigorous selection protocol the models of Occam’s group all provide a parsimonious description of the data. This cannot be guaranteed by simply ranking all candidate models by their AIC.

For heart diseases, the procedure was analogous. When the full Preston baseline model was combined with the LNT model and fitted as an ERR-LNT model to the data, it yielded a final deviance of 13,148.55. This model contains 30 parameters (29 baseline parameters plus the slope of the LNT model). Subsequently, all baseline parameters were tested for their significance using the LRT. Rigorous testing led to a new set of statistically significant baseline parameters with nine parameters less than the full Preston baseline model (see Online Resource, Table S7). This streamlined baseline model was combined with the dose–response models from Fig. 1 as ERR models and fitted to the data. The same selection protocol involving the LRT was then applied to compare nested dose–response models with each other and to eliminate those nested models with inferior deviance values (see pages 18 and 19 of the Online Resource together with Figure S1 and Table S5 for details). For each surviving dose–response model, the three DEMs (sex, attained age and age at exposure) were tested for statistical significance. Subsequently, the full Preston baseline model was combined with an LNT model and implemented as EAR-LNT model yielding dev = 13,155.96. The streamlining process led to the same baseline model as the one obtained in combination with the ERR-LNT model (see Online Resource, Table S7). Then this streamlined baseline model was combined with the models from Fig. 1 as EAR models and fitted to the data. Pages 19 and 20 of the Online Resource together with Figure S1 and Table S6 provide all necessary details related to the selection of the final non-nested EAR models. Altogether, these selections lead to a set of final non-nested models that have been used for MMI.

### Statistical analyses

The MECAN software (Kaiser 2010) was applied to fit the EAR and ERR models to the data, using Poisson regression to estimate the values of the adjustable model parameters by fitting the model to the grouped data. For the minimization of the deviance, MECAN applies the MINUIT package for function minimization (Moneta and James 2010). The *ERR* and *EAR* estimates are calculated directly from *h* and *h*_0_. Confidence intervals (CI) for the *ERR* and *EAR* estimates (both, for the final non-nested models that are included into Occam’s group and for MMI) were simulated using multi-variate normal distributions for parameter uncertainties that obey the parameter correlation matrix (Kaiser and Walsh 2013). For a risk variable such as *ERR*, a probability density distribution of 10^4^ realizations is generated, which is used to estimate 95% CI. Central risk estimates were calculated from the maximum likelihood estimates (MLEs) of the model parameters. The MECAN package and all model-related input and result files are available from the authors.

Specifically, for each final non-nested model the Akaike Information Criterion (AIC) (Akaike 1973, 1974) is calculated: AIC = dev + 2 × *N*_*par*_, where *N*_*par*_ is the number of model parameters. Models with smaller values of AIC are favored based on fit (via dev) and parameter parsimony (models with more parameters get punished by the factor 2 × *N*_*par*_) (Walsh 2007). The AIC is essential for MMI: For a set of final non-nested models, the AIC-weights are calculated, which are proportional to exp(− 0.5 AIC), i.e. models with smaller AIC are assigned a larger weight (see Online Resource, page 10). The resulting weights, multiplied by a factor of 10^4^, give the number of samples for risk estimates to be generated by uncertainty distribution simulations. Then, the created model-specific probability density functions are merged. The resulting probability density distribution represents all uncertainties arising from the different models and their superposition. Central risk estimates from MMI are calculated from the AIC-weighted MLEs for single risk models. 95% CI are derived from the final merged MMI probability density distribution.

## Results

### Cerebrovascular diseases

**Fig. 2:**
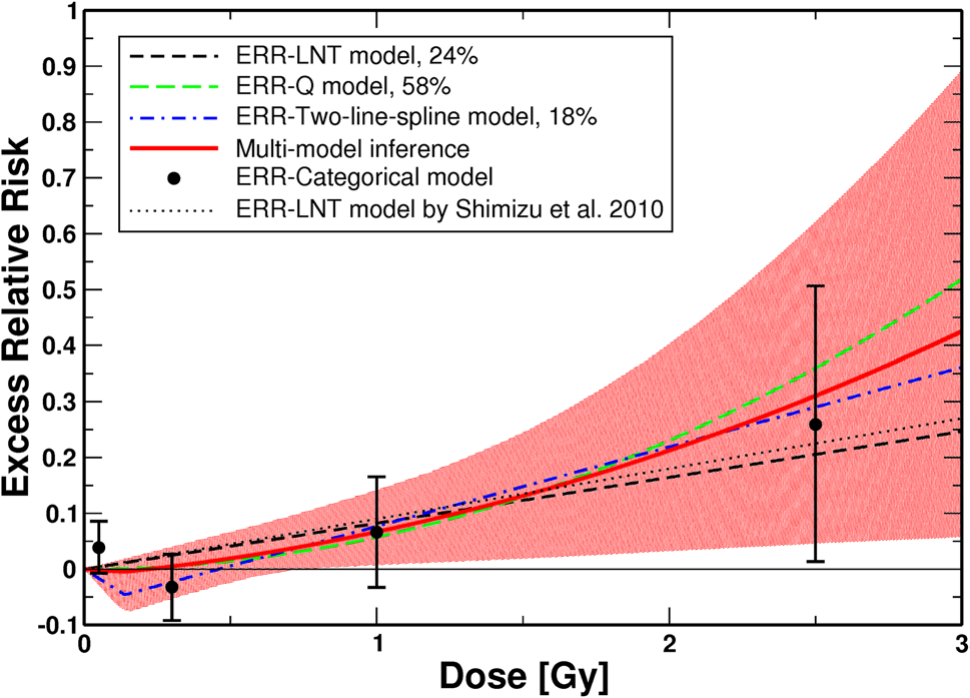
*ERR* for mortality from CeVD versus weighted colon dose (full dose range) for the three final non-nested ERR models and the simulated dose–response curve from MMI. The shaded area represents the 95% CI region for the MMI. For AIC-weights see insert. The dotted line shows the prediction from Shimizu et al. (2010). Point estimates and related 95% CI from the fit of a categorical ERR model that divides the dose range into the following categories (*D* < 0.005 Gy, 0.005 Gy ≤ *D* < 0.1 Gy; 0.1 Gy ≤ *D* < 0.5 Gy, 0.5 Gy ≤ *D* < 1.5 Gy, and *D* ≥ 1.5 Gy) are shown. In the categorical fit, zero risk was assigned to the dose range *D* < 0.005 Gy. The point estimates from the categorical model are provided for comparison. The figure is valid for men and women of both cities. Online version contains color

For mortalities from CeVD three non-nested ERR models survived the selection protocol: the ERR-LNT, ERR-Q and ERR-two-line spline models. They were used for MMI. None of the DEMs were significant. For these three models Table 1 provides all essential information obtained by fitting them to the data. For the related model parameters (baseline and radiation-associated), their MLEs and symmetric, Wald-type standard errors, see Table S3 in the Online Resource. Figure 2 shows the dose–responses for *ERR* for the three models and the dose–response curve from MMI together with point estimates and related 95% CI from a categorical ERR model. The three models were stable under cross-validation (Stone 1977; Kohavi 1995) using 10, 20, 40 and 80 folds. For the ERR-two-line spline model some of the cross-validations may have led to *ERR* < − 1 (which implies negative values for the hazard) so that this model showed a somewhat reduced stability under cross-validations. For risk values at various preselected values of age at exposure and attained age, see Online Resource, Table S4.

**Table 1:** For both detrimental health outcomes (mortality from CeVD and heart diseases) the final non-nested models that are included into Occam’s group are shown with their final deviances (dev), difference in final deviances (Δdev) with respect to the model with the smallest final deviance, number of model parameters (*N*_*par*_), AIC-values, difference in AIC-values (ΔAIC) with respect to the model with the smallest AIC-value, and AIC-weights

	dev	Δdev	*N*_*par*_	AIC	ΔAIC	AIC-weight
CeVD (ICD-9 430–438)						
Streamlined baseline model	13,422.27		15	13,452.27		
ERR-LNT model	13,417.53	3.38	16	13,449.53	1.76	0.2412
ERR-Q model	13,415.77	1.61	16	13,447.77	0	0.5823
ERR-two-line spline model, *D*_*th*_ = 0.14 Gy	13,414.15	0	18	13,450.15	2.39	0.1765
Heart diseases (ICD-9 393–429, excluding 401, 403, 405)						
Streamlined baseline model	13,163.17		20	13,203.17		
ERR-LNT model	13,152.52	1.29	21	13,194.52	0	0.3089
ERR-Q model	13,154.54	3.32	21	13,196.54	2.02	0.1123
ERR-smooth step model, *D*_*th*_ = 1.52 Gy	13,153.66	2.44	23	13,199.66	5.14	0.0236^b^
EAR-LNT model^a^	13,151.22	0	22	13,195.22	0.71	0.2171
EAR-Q model^a^	13,151.78	0.55	22	13,195.78	1.26	0.1647
EAR-LTH model, *D*_*th*_ = 2.36 Gy	13,152.29	1.07	22	13,196.29	1.78	0.1271
EAR-smooth step model, *D*_*th*_ = 2.54 Gy	13,152.31	1.09	23	13,198.31	3.79	0.0464^b^

The Akaike Information Criterion is denoted by AIC (AIC = dev + 2 × *N*_*par*_). For CeVD, the deviance related to MMI is 13,415.46. As a comparison, the results from fitting the streamlined baseline models are also provided

^a^Contains an age-dependent dose–effect modifier

^b^See Online Resource, page 10, for an explanation why for heart diseases models with AIC-weights < 0.05 were included into the set of non-nested models that was used for MMI

**Fig. 3:**
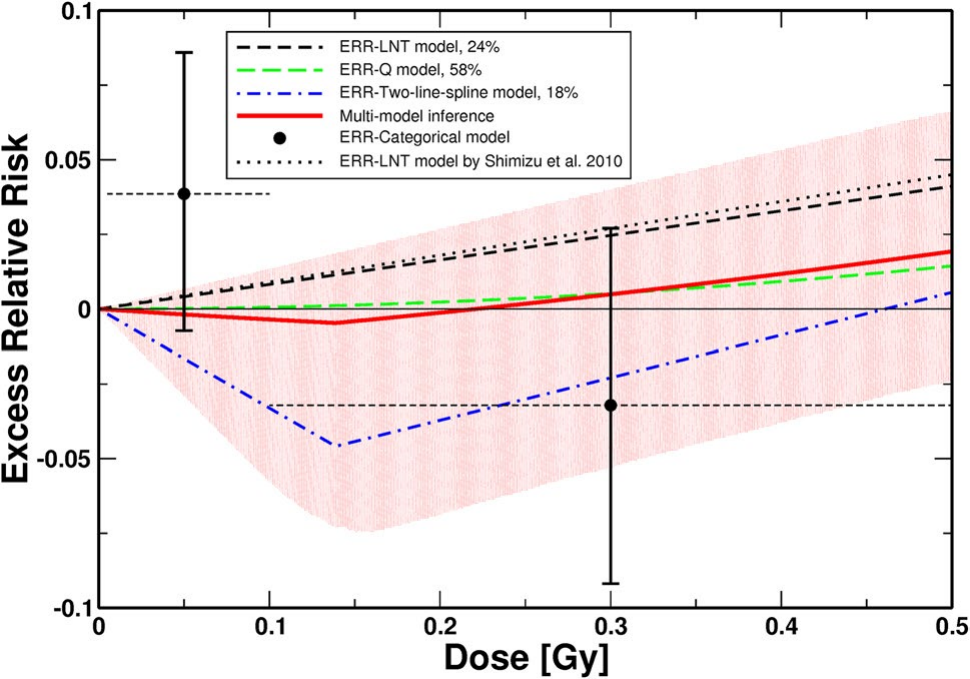
*ERR* for mortality from CeVD versus weighted colon dose (lower dose range) for the three final non-nested ERR models and the simulated dose–response curve from MMI. The shaded area represents the 95% CI region for the MMI. For AIC-weights see insert. The dotted line shows the prediction from Shimizu et al. (2010). Point estimates and related 95% CI from the fit of a categorical ERR model that divides the dose range into the following categories (*D* < 0.005 Gy, 0.005 Gy ≤ *D* < 0.1 Gy; 0.1 Gy ≤ *D* < 0.5 Gy, 0.5 Gy ≤ *D* < 1.5 Gy, and *D* ≥ 1.5 Gy) are shown (the dose categories are indicated by horizontal dashed lines). In the categorical fit, zero risk was assigned to the dose range *D* < 0.005 Gy. The point estimates from the categorical model are provided for comparison. The figure is valid for men and women of both cities. Online version contains color

For doses smaller than approximately 1.4 Gy the MMI predicts a risk lower than the one based on the LNT model (Fig. 2). The potentially protective effects visible in Fig. 3 stem from the 18% contribution of the two-line spline model, which contains an inflection point at 0.14 Gy (see Table 1 and Online Resource, Table S3). The protective effects are, however, not statistically significant because the upper bounds of the 95% CI for MMI include zero risk. The results from MMI imply that up to 0.75 Gy no inference can be drawn about the radiation risk because the MMI-related 95% CI include zero risk up to that dose (Fig. 2). For doses larger than 1.5 Gy the LNT model underestimates the risk compared to the dose response from MMI (Fig. 2). This is also visible from Table 2, which shows the radiation-associated excess cases calculated according to the three final non-nested models and according to MMI. At higher doses the LNT model predicts a lower number of excess CeVD cases compared to MMI.

**Table 2:** Radiation-associated excess cases for CeVD according to the three final non-nested models and MMI

Dose-bin	ERR-LNT	ERR-Q	ERR-two-line spline model	MMI
0–0.005 Gy	0.3	0	− 1.4	− 0.2
0.005–0.06 Gy	4.9	0.1	− 20	− 2.3
0.06–0.1 Gy	3.5	0.2	− 14.1	− 1.5
0.1–0.25 Gy	11.8	1.4	− 35.7	− 2.6
0.25–0.5 Gy	15.9	4	− 8.2	4.7
0.5–1 Gy	19.8	9.9	11.6	12.6
1–1.5 Gy	12	10.4	13	11.2
1.5–2 Gy	5.5	6.7	7	6.5
2–2.5 Gy	5.3	8.6	7.5	7.6
2.5–3 Gy	3.1	5.8	4.5	4.9
3 Gy–	0.1	0.1	0.1	0.1
Sum	82.2	47.2	− 35.7	41.0

Negative “excess” cases indicate a protective effect

### Heart diseases

**Fig. 4:**
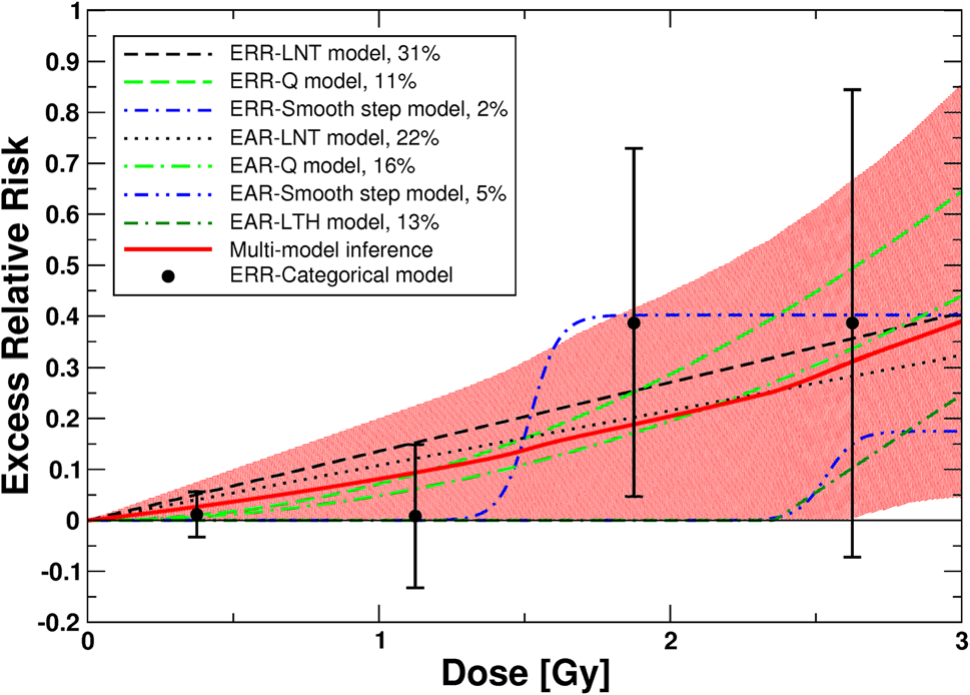
*ERR* for mortality from heart diseases versus weighted colon dose for the seven final non-nested ERR and EAR models and for the simulated dose–response curve from MMI. The shaded areas represent the 95% CI region for the MMI. For AIC-weights see insert. Also provided are point estimates and related 95% CI from the fit of a categorical ERR model that divides the dose range into the following categories (*D* < 0.005 Gy, 0.005 Gy ≤ *D* < 0.75 Gy; 0.75 Gy ≤ *D* < 1.5 Gy, 1.5 Gy ≤ *D* < 2.25 Gy, and *D* ≥ 2.25 Gy) with zero risk assigned to the dose range *D* < 0.005 Gy. The point estimates from the categorical model are provided for comparison. The figure is valid for males from Hiroshima. The preselected values for age at exposure and attained age are 30 and 70 years, respectively. For correction factors for city and females, see Table S8 in the Online Resource. Online version contains color

**Fig. 5:**
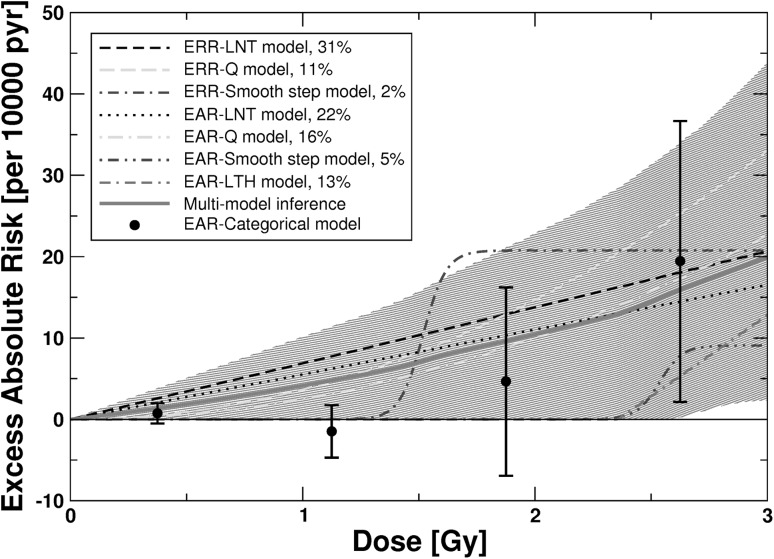
*EAR* for mortality from heart diseases versus weighted colon dose for the seven final non-nested ERR and EAR models and for the simulated dose–response curve from MMI. The shaded areas represent the 95% CI region for the MMI. For AIC-weights see insert. Also provided are point estimates and related 95% CI from the fit of a categorical EAR model that divides the dose range into the following categories (*D* < 0.005 Gy, 0.005 Gy ≤ *D* < 0.75 Gy; 0.75 Gy ≤ *D* < 1.5 Gy, 1.5 Gy ≤ *D* < 2.25 Gy, and *D* ≥ 2.25 Gy) with zero risk assigned to the dose range *D* < 0.005 Gy. This EAR-categorical model contains an age-dependent DEM. The point estimates from the categorical model are provided for comparison. The figure is valid for males from Hiroshima. The preselected values for age at exposure and attained age are 30 and 70 years, respectively. For correction factors for city and females, see Table S8 in the Online Resource. Online version contains color

For heart diseases, the final non-nested models in Occam’s group consisted of ERR models (LNT, Q, smooth step) and EAR models (LNT, Q, LTH, smooth step). They were used for MMI. For these seven models Table 1 provides all essential information obtained by fitting them to the data. For the related model parameters (baseline and radiation-associated), their MLEs and symmetric, Wald-type standard errors, see Online Resource, Table S7. The fit with the smallest AIC (i.e. ΔAIC = 0) was achieved by the ERR-LNT model (Table 1). The EAR-LNT and EAR-Q models contain age-dependent DEMs (see Online Resource, Table S7). Figure 4 shows the dose–responses for *ERR* for the seven non-nested models and the dose–response curve from MMI together with point estimates and related 95% CI from a categorical ERR model. The *EAR* versus dose is provided in Fig. 5. The seven models were stable under cross-validation using 10, 20, 40, and 80 folds. For risk values at various preselected values of age at exposure and attained age, see Online Resource, Table S8.

The dose responses from MMI contain mostly contributions from the linear and quadratic models (Table 1). Up to 2.58 Gy no conclusive prediction can be given about the radiation risk because the MMI-related 95% CI include zero risk up to that dose (Figs. 4, 5). The ERR-LNT model overpredicts the risk compared to the prediction from MMI (Figs. 4, 5). This is also visible from Table 3, which shows the radiation-associated excess cases calculated according to the seven final non-nested models and according to MMI. Up to 2.5 Gy the ERR-LNT model predicts a higher number of excess cases from heart diseases compared to MMI.

**Table 3:** Radiation-associated excess cases for heart diseases according to the seven final non-nested models and MMI

Dose-bin	ERR-LNT	ERR-Q	ERR-smooth step	EAR-LNT	EAR-Q	EAR-LTH	EAR-smooth step	MMI
0–0.005 Gy	0.5	0	0	0.4	0	0	0	0.2
0.005–0.06 Gy	7.2	0.1	0	5.7	0	0	0	3.5
0.06–0.1 Gy	5	0.2	0	3.9	0.1	0	0	2.4
0.1–0.25 Gy	17.1	1.6	0	13.1	1	0	0	8.5
0.25–0.5 Gy	23.1	4.5	0	18.2	3	0	0	12.1
0.5–1 Gy	28.5	10.9	0	23.9	8.1	0	0	16.5
1–1.5 Gy	17.7	11.6	1	14.8	8.7	0	0	11.4
1.5–2 Gy	7.7	7	12.1	6.8	5.7	0	0	5.9
2–2.5 Gy	7.7	9.3	10.2	7	7.7	0	0.1	6.5
2.5–3 Gy	4.5	6.3	5.1	4	5.2	4.9	6.1	4.8
3 Gy–	0.1	0.1	0.1	0.1	0.3	1.5	0.8	0.3
Sum	119.10	51.60	28.50	97.90	39.80	6.40	7.00	72.2

## Discussion

In the present study the cohort of the latest LSS data for CVD was analyzed including the full follow-up period from 1950 to 2003 with proximal and distal survivors, in line with the comments by Little et al. (2013) and the findings by Schöllnberger et al. (2015) related to the absence of a healthy survivor selection effect for CeVD and heart diseases in these data. For each detrimental health outcome one set of final non-nested models was found that all describe the data approximately equally well (Table 1). The variety of dose–responses in these two sets may reflect the heterogeneity of the diseases subsumed under CeVD and heart diseases. For such situations MMI can be applied with some confidence as long as the candidate models have been chosen with care, reflecting as many biologically plausible shapes for dose–responses as possible. Here, linear, supralinear and sublinear models were allowed for (Fig. 1) so that it can be expected that MMI was a suitable method in the current analysis. This is in accordance with the findings of Furukawa et al. (2015) who simulated various data sets using one specific dose–response. For all solid cancers they found that MMI performed well when the model that had been used to simulate the data sets was also included in the candidate models. As in the present study, the endpoint of all solid cancers comprises several (organ-specific) endpoints with potentially different dose responses. In that context it is interesting to note that for CeVD with its lower number of aggregated diseases compared to heart diseases MMI finds only three final non-nested models compared to the seven for heart.

For CeVD, the ERR-Q model provides the best fit (Table 1), as already found by Shimizu et al. (2010). The two other models fit the data almost equally well (Table 1) and are also included in the MMI, resulting in an upwardly curved dose–response with a shallow dip below zero risk at low doses (Figs. 2, 3). This area of negative risk stems from the 18% contribution of the two-line spline model. This protective effect is, however, not statistically significant because the 95% CI for the MMI include zero risk.

While the dose–response from MMI is consistent with a threshold-dose of 0.2 Gy (Fig. 3), the upper bound of the related 95% CI includes positive values. Given that the lower bound of the 95% CI includes zero risk up to a dose of 0.75 Gy (Fig. 2), our findings imply that it cannot be concluded whether there is a risk up to that dose. The result from applying the ERR-two-line spline model finds confirmation by a fit with a categorical model (Figs. 2, 3). It is interesting to note that the dose–response from the two-line spline model (after exhibiting the minimum at 0.14 Gy, it crosses the *x*-axis at 0.46 Gy) is consistent with *D*_*th*_ = 0.48 Gy from the ERR-LTH model and with the findings from testing the ERR-hormesis model. The latter, like the ERR-LTH model not statistically significant, exhibits its no-observed-adverse-effect level (NOAEL) (Duffus et al. 2007) at 0.55 Gy.

In Table 2 the radiation-associated cases are provided for the three final non-nested models and MMI, which leads to some negative “excess” cases at low doses, indicating a small protective effect in accordance with the dose–response in Figs. 2 and 3. Altogether, MMI predicts only approximately half as many deaths as the LNT model (Table 2). That is consistent with Fig. 2, which shows that up to approximately 1.4 Gy the LNT model predicts a higher risk than the dose–response from MMI and because most cases are associated with lower doses up to 0.25 Gy. Mitchel et al. (2011) report low-dose induced protective anti-inflammatory effects in atherosclerosis prone ApoE^−/−^ mice that were confirmed by other researchers (Le Gallic et al. 2015; Mathias et al. 2015). Mitchel et al. (2011) state that their results were distinctly non-linear with dose with maximum protective effects tending to occur at 25 or 50 mGy.

For heart diseases, a mixture of ERR and EAR models contributes to MMI with the strongest contributions from LNT (53%) and Q models (28%) (Table 1). Consequently, the dose–response from MMI is approximately linear-quadratic and predicts a lower risk over the whole dose range compared to the ERR-LNT model (Figs. 4, 5). This finds confirmation by the results in Table 3: For each dose-segment MMI predicts a lower number of excess cases than the LNT model. Interestingly, the lower bound of the MMI-related 95% CI is zero up to 2.58 Gy, implying that it cannot be concluded whether there is a risk up to that dose.

The risk predictions for the EAR-LTH and the EAR-smooth step models in Fig. 5 are independent of the attained age. The EAR-LNT and EAR-Q models, however, contain attained age dependent DEMs. Risk predictions of age-independent EAR models are strongly influenced by younger ages. For attained ages < 70 years the EAR-LNT and EAR-Q models are multiplied by a factor < 1 due to the attained age-dependent DEM (see Online Resource, Table S7). The *EAR*-predictions in Fig. 5 for the three ERR models are valid for an attained age of 70 years because *EAR* = *h*_0_ *ERR*; for ages < 70 years the age-dependent baseline hazard *h*_0_ decreases and so do the *EAR*-predictions. The curves in Fig. 5 are, therefore, consistent with each other. Analogous considerations apply for Fig. 4. We hypothesize that the different shapes visible in Figs. 4 and 5 reflect the large biological differences in endpoints subsumed under “heart diseases” (refer to “Materials and methods” section). The EAR-LTH model, e.g. predicts only six excess cases, while according to the ERR-LNT model there should be 119 radiation-associated cases (Table 3). It is anticipated that the different dose responses each describe one subgroup of endpoints within the heart diseases. For CeVD, Table 2 provides the radiation-associated excess cases predicted by the different final models and by MMI. Because most cases are located at rather low doses (mean dose of cases = 111 mGy), where the two-line spline model exhibits its dip below zero risk (Fig. 3), it is plausible that this model predicts a negative number of “excess cases” (Table 2). The ERR-Q model predicts a very small excess risk for CeVD mortality at low doses (Fig. 3 and Online Resource, Table S4). Consequently, fewer excess cases are associated with that model compared to the ERR-LNT model (47 versus 82, Table 2).

Additional analyses with altered data sets that contain smaller dose-bins revealed that the nonlinearities of the final non-nested models do not depend on the dose stratification of the grouped LSS data.

When applying the Bayesian information criterion (BIC) (Claeskens and Hjort 2008) to calculate the BIC-weights (analogous as in Eq. (S2) of the Online Resource) one obtains the following values: 0.2929 (ERR-LNT model), 0.7070 (ERR-Q model) and 0.000165 (ERR-two-line spline model). It is BIC = dev + *N*_*par*_ × ln(*n*), where *N*_*par*_ is the number of model parameters and *n* is the number of observations, i.e. CeVD mortality cases: *n* = 9622. The reason for the very small BIC-weight for the two-line spline model is the well-known feature of the BIC to penalize additional parameters stronger than the AIC as long as the number of observations is large. The two-line spline model with its additional two parameters (Table 1) consequently performs worse under this criterion compared to its performance under the AIC. The resulting dose–response from MMI is essentially a linear-quadratic model with the strongest contribution from the ERR-Q model (result not shown). Similar results are obtained for heart diseases (BIC-weights for the ERR-LNT and ERR-Q models are 0.708 and 0.2574, respectively).

Additional analyses showed that our results are fairly independent of the chosen baseline model. When the full Preston baseline model in combination with the different dose–response models was applied to the data for CeVD, very similar values for the AIC-weights were found compared to those reported in Table 1. For heart diseases, small differences in the values of Δdev and ΔAIC were detected, resulting in small differences in the AIC-weights of a few percent. We prefer parsimonious models because they are more stable, lead to less uncertainty in the risk estimates and contain less parameter correlations compared to models with a larger number of parameters.

The present study extends the analysis of Shimizu et al. (2010) for the same data set in an important manner. They tested four ERR models (LNT, Q, LQ, LTH) that were combined with a stratified baseline model. Here, a further eight dose–response models were tested (Fig. 1) allowing for various nonlinear dose–responses. All models were applied as ERR and EAR models in combination with a parametric baseline model. The most important innovation relates to the description of the dose–response with several models in contrast to relying on a single model of choice. MMI allows dose–response relationships to be weighted averages of different underlying mathematical functions. Given the grouping of multiple endpoints under CeVD and heart diseases, real dose–responses might not be exactly linear. On the other hand, MMI might not cover important features of the dose–response, if the “true” model is very different from the candidate models (Furukawa et al. 2015). We tried to control for this case by applying a large number of plausible candidate models. For CeVD, Shimizu et al. (2010) applied the LNT model for their risk predictions and obtained *ERR* = 0.09 (0.01, 0.17) per Gy. This is consistent within the reported errors with the risk prediction from MMI at 1 Gy: *ERR* = 0.0669 (0.0074, 0.14) (see Online Resource, Table S4). Our risk prediction is somewhat lower mostly because of the 58% contribution from the ERR-Q model (Fig. 2). Yet, the most important difference appears at lower doses: Here, the MMI-based analysis implies that it cannot be concluded whether there is a risk up to 0.75 Gy (Fig. 2). Shimizu et al. (2010) did not find evidence for a threshold: Their best estimate and 95% CI of a threshold-dose for CeVD was 0.5 Gy (≤ 0, ∼ 2). For heart diseases, Shimizu et al. (2010) report that a linear model fitted the data better than a dose-squared model. Their risk prediction of *ERR* = 0.14 (0.06, 0.23) is consistent within the reported errors with the risk prediction from MMI at 1 Gy: *ERR* = 0.08 (0, 0.20) (see Table S8 in the Online Resource). The lower bound of the 95% CI, however, is zero up to a dose of 2.6 Gy (Figs. 4, 5). For both aggregate detrimental health outcomes MMI implies a borderline significance for the risk below doses of 0.75 and 2.6 Gy, respectively. In marked contrast, risk coefficients from LNT models are determined mainly by effects at higher doses but inevitably predict significant risk increases even at lowest doses. It is interesting to note that Dr. Furukawa has recently analyzed the data by Shimizu et al. (2010) using a Bayesian semiparametric approach as in his analysis of all solid cancers (Furukawa et al. 2015). For both detrimental health outcomes (CeVD and heart diseases) he found very similar dose–responses compared to the dose–responses from MMI in Figs. 2 and 4 (personal communication of coauthor Dr. J.C. Kaiser with Dr. K. Furukawa).

The current study also poses a major enhancement compared to Schöllnberger et al. (2012) who analyzed a smaller cohort with follow-up from 1968 to 1997 restricted to proximal survivors. The updated cohort includes approximately twice as many deaths from CeVD and heart diseases and almost three times as many person-years. To avoid unrealistic steep slopes and sharp edges the current study applied a range of biologically realistic smooth dose responses including a hormesis model (Fig. 1), which had been introduced by Brain and Cousens (1989) to describe stimulation of plant growth after low-dose herbicide exposures. Motivation for allowing a larger range of different dose responses can be found in the biological literature. Dose responses that allow for protective effects at low doses such as the LQ model, hockey-stick, hormesis and two-line spline models can be justified from the work of Mitchel et al. (2011, 2013). These authors found U-shaped and J-shaped dose–responses in mice for endpoints associated with CVD. Anti-inflammatory effects that play an important role in that context are currently intensely studied (see, e.g. the reviews by Rödel et al. (2012a, b)) and have been reported by Le Gallic et al. (2015) and Mathias et al. (2015). Earlier, Mitchel et al. (2007) showed that low doses of γ-radiation delivered at low dose rates exhibit a protective effect related to chronic ulcerative dermatitis, an inflammatory skin reaction, in C57BL/6 mice, decreasing both disease frequency and severity and extending the life span of older animals. LTH models are another realistic possibility for dose responses related to radio-epidemiological cohorts given the findings of Mitchel et al. (2011, 2013) and Le Gallic et al. (2015) on low-dose induced protective anti-inflammatory effects. Mathias et al. (2015) provided evidence for anti-inflammatory effects after low-dose exposure but also found some pro-inflammatory responses. In such a situation a LTH model may describe a data set better than the LNT model. This finds confirmation in the study by Mitchel et al. (2007) who state that their dermatitis data indicate that low doses may generally produce either no effect or protective effects with respect to this autoimmune- and age-related non-cancer disease in mice. The findings of anti-inflammatory protective effects at low doses and detrimental effects at moderate (0.3 Gy) and higher doses (6 Gy) (Mancuso et al. 2015) provide the biological context for applying the smooth step and the step-linear models (Fig. 1). A step-type response (with a steep slope, Fig. 1) may reflect the distinct dose at which protective mechanisms are lost. Different tissues and different individuals can be expected to have different threshold-doses, leading to an overall smooth transition. While at low doses it is feasible that risk increase may be balanced by a protective decrease as in the LTH model, a smooth transition zone may exist where risk increases steadily, followed by a linear increase in risk at even higher doses, as in the step-linear model.

For CeVD, the ERR-two-line spline model crosses the *x*-axis at 0.46 Gy. This together with the fact that up to 0.75 Gy no reliable conclusions can be drawn about the radiation risk (Figs. 2, 3) is overall consistent with the result by Schöllnberger et al. (2012). At 1 Gy, however, risk estimates are about 2.5 times smaller compared to our previous study due to the exclusion of the biologically implausible step model. While the results for heart diseases are overall consistent with the results from Schöllnberger et al. (2012), the new results are more reliable because of the much larger number of cases in the Shimizu et al. (2010) data and due to the applied smooth dose–response models. Sublinear dose–responses have also been found for other cohorts, such as, e.g. for CeVD incidence in the Mayak Workers Cohort (Simonetto et al. 2015) and for ischemic heart diseases in the Canadian Fluoroscopy Cohort Study (Helmut Schöllnberger, Jan Christian Kaiser, Markus Eidemüller, Lydia Zablotska. Dose–responses for mortality from ischemic heart diseases in the Canadian Fluoroscopy Cohort: 1950–1987. In preparation). Takahashi et al. (2012) found a threshold-dose of 1.3 Gy for female A-bomb survivors in the Adult Health Study.

The aggregate detrimental health outcomes analyzed in the present study could in principle be separated into single data sets, each of them related to one single disease. One could then test the 13 dose–response models that were applied in the present study on these data sets separately and obtain single dose–responses, one for each disease. From these single dose–responses a case-weighted mean dose–response could be calculated. Such an analysis is out of the scope of the present study but the result should be close to the dose–responses from MMI depicted in Figs. 2, 3, 4 and 5. The technique of MMI is an elegant and efficient way to perform such analyses.

Recent analyses highlight the controversial nature of ongoing discussions related to the shape of dose responses for CVD (Little 2016). Our analyses lead to sublinear dose responses and tendentially to lower risk predictions at low doses compared to the study by Little (2016). We advocate careful analyses of each single cohort in contrast to meta-analyses under the assumption of LNT dose responses (Little et al. 2012; Little 2016). This recommendation also holds for other detrimental health effects such as cancer.

## Conclusions

The present analysis shows a sublinear dose–response for mortalities from CeVD at low and medium doses (0–1.4 Gy). At higher doses the LNT model underestimates the risk compared to the dose response from MMI. Similarly, for heart diseases, a sublinear dose–response was found as well (0–3 Gy). Our analysis appeals to the more complex picture that arises from analyzing aggregate endpoints and their possibly different radiobiological mechanisms. Together with the sublinearity this may be a hint that different biological mechanisms may operate at low and medium doses compared to high doses. Our study provides an elegant way to analyze radioepidemiological data sets, which comprise a number of similar end points. The MMI method can similarly be applied to other aggregate health outcomes with aggregated endpoints such as all solid cancers or all leukaemias. Because the internationally applied guidelines for radiation protection largely rely on analyses of the LSS data and the LNT model, our findings have important implications for risk assessment of IR in the context of medical applications (such as CT scans, radiotherapy and low-dose anti-inflammatory radiotherapy), nuclear energy production and accident related long-term risks.

## Electronic supplementary material

Below is the link to the electronic supplementary material.

Supplementary material 1 The Online Resource provides in Table S1 the number of individuals and death cases from CeVD and heart diseases stratified by sex, age at exposure and weighted colon dose. Subsequently, the mathematical form of the baseline model that had been applied by Preston et al. (2003) is presented. Pages 6 to 8 give the mathematical form of all dose-response models that were tested in the present study. Figure S1 provides the number of model parameters for the applied dose-response models and relation between the models regarding their nestedness. Page 10 contains the section “Calculation of AIC-weights.” It supplies an equation that was used to calculate the AIC-weights given in Table 1 (main text) together with additional explanations. The next section gives a detailed description of how the model selection was performed for the CeVD mortality data. Table S2 provides the results of fitting the dose-response models from Figure 1 as ERR models to the mortality data for CeVD; among other information, the final deviance values are provided together with the AIC-values. Table S3 supplies model parameters (baseline and radiation-associated), maximum likelihood estimates and Wald-type standard errors for the three final non-nested models that were used for MMI for the CeVD mortality data. Table S4 supplies values for *ERR* and *EAR* for mortality from CeVD at 0.1 and 1 Gy calculated with MMI and with the three final non-nested models that were used for MMI. This is followed by a short mathematical derivation of correction factors that allow obtaining city-averaged *EAR*-values for Table S4. Pages 18 to 20 provide complementary information regarding the model selection for the mortality data for heart diseases. Table S5 provides the results of fitting the dose-response models from Figure 1 as ERR models to the mortality data for heart diseases; among other information, the final deviance values are shown together with the AIC-values. Table S6 supplies analogous information as Table S5 but with the dose-response models implemented as EAR models. Table S7 shows model parameters (baseline and radiation-associated), maximum likelihood estimates and Wald-type standard errors for the seven final non-nested models that were used for MMI for the heart diseases mortality data. Table S8 supplies values for *ERR* and *EAR* for mortality from heart diseases at 1 Gy calculated with MMI and with the seven final non-nested models that were used for MMI. This is followed by an explanation of how to calculate correction factors for city and females to be used in Table S8. (DOC 460 KB)
