# Correction to: Dose–responses for mortality from cerebrovascular and heart diseases in atomic bomb survivors: 1950–2003

**DOI:** 10.1007/s00411-019-00779-0

**Published:** 2019-02-24

**Authors:** Helmut Schöllnberger, Markus Eidemüller, Harry M. Cullings, Cristoforo Simonetto, Frauke Neff, Jan Christian Kaiser

**Affiliations:** 1 Helmholtz Zentrum München, Department of Radiation Sciences, Institute of Radiation Protection, Ingolstädter Landstrasse 1, 85764 Neuherberg, Germany; 2 Department of Radiation Protection and the Environment, Federal Office for Radiation Protection, Ingolstädter Landstrasse 1, 85764 Neuherberg, Germany; 3 Department of Statistics, Radiation Effects Research Foundation, Hiroshima, Japan; 4 Institute of Pathology, Städtisches Klinikum München and Technical University of Munich, Munich, Germany

## Correction to: Radiation and Environmental Biophysics (2018) 57(1):17–29. 10.1007/s00411-017-0722-5

The article Dose–responses for mortality from cerebrovascular and heart diseases in atomic bomb survivors: 1950–2003, written by Helmut Schöllnberger, Markus Eidemüller, Harry M. Cullings, Cristoforo Simonetto, Frauke Neff, Jan Christian Kaiser, was originally published electronically on the publisher’s internet portal (currently SpringerLink) on 8 December 2017 without open access. With the author(s)’ decision to opt for Open Choice the copyright of the article changed on 24 February 2019 to © The Author(s) 2019 and the article is forthwith distributed under the terms of the Creative Commons Attribution 4.0 International License (http://creativecommons.org/licenses/by/4.0/), which permits use, duplication, adaptation, distribution and reproduction in any medium or format, as long as you give appropriate credit to the original author(s) and the source, provide a link to the Creative Commons license and indicate if changes were made.

