# Supplementary material for: Dose-responses for mortality from cerebrovascular and heart diseases in atomic bomb survivors: 1950–2003
Source: Radiat Environ Biophys. 2017 Dec 8;57(1):17–29. doi: 10.1007/s00411-017-0722-5 (PMC6373359; doi:10.1007/s00411-017-0722-5)
Supplement: Supplementary file 1 — Supplementary material 1 The Online Resource provides in Table S1 the number of individuals and death cases from CeVD and heart diseases stratified by sex, age at exposure and weighted colon dose. Subsequently, the mathematical form of the baseline model that had been applied by Preston et al. (2003) is presented. Pages 6 to 8 give the mathematical form of all dose-response models that were tested in the present study. Figure S1 provides the number of model parameters for the applied dose-response models and relation between the models regarding their nestedness. Page 10 contains the section “Calculation of AIC-weights.” It supplies an equation that was used to calculate the AIC-weights given in Table 1 (main text) together with additional explanations. The next section gives a detailed description of how the model selection was performed for the CeVD mortality data. Table S2 provides the results of fitting the dose-response models from Figure 1 as ERR models to the mortality data for CeVD; among other information, the final deviance values are provided together with the AIC-values. Table S3 supplies model parameters (baseline and radiation-associated), maximum likelihood estimates and Wald-type standard errors for the three final non-nested models that were used for MMI for the CeVD mortality data. Table S4 supplies values for ERR and EAR for mortality from CeVD at 0.1 and 1 Gy calculated with MMI and with the three final non-nested models that were used for MMI. This is followed by a short mathematical derivation of correction factors that allow obtaining city-averaged EAR-values for Table S4. Pages 18 to 20 provide complementary information regarding the model selection for the mortality data for heart diseases. Table S5 provides the results of fitting the dose-response models from Figure 1 as ERR models to the mortality data for heart diseases; among other information, the final deviance values are shown together with the AIC-values. Table S6 supplies analogous [file 411_2017_722_MOESM1_ESM.doc]

**Supplementary Material**

**Dose-responses for mortality from cerebrovascular and heart diseases in atomic bomb survivors: 1950-2003**

Radiation and Environmental Biophysics

Helmut Schöllnberger, Markus Eidemüller, Harry M. Cullings, Cristoforo Simonetto, Frauke Neff, Jan Christian Kaiser

Helmut Schöllnberger (🖂)

Helmholtz Zentrum München

Department of Radiation Sciences

Institute of Radiation Protection

Ingolstädter Landstrasse 1

D-85764 Neuherberg

Germany

Fax: +49 89-3187-3363

Email: [schoellnberger@helmholtz-muenchen.de](mailto:schoellnberger@helmholtz-muenchen.de)

Federal Office for Radiation Protection

Department of Radiation Protection and the Environment

Ingolstädter Landstrasse 1

D-85764 Neuherberg

Germany

Email: [hschoellnberger@bfs.de](mailto:hschoellnberger@bfs.de)

**Table of Contents**

[Table S1 3](#__RefHeading___Toc493965437)

[Preston baseline model 4](#__RefHeading___Toc493965438)

[Mathematical functions used for the 13 parametric dose-response models from Figure 1 6](#__RefHeading___Toc493965439)

[Figure S1 9](#__RefHeading___Toc493965440)

[Calculation of AIC-weights 10](#__RefHeading___Toc493965441)

[Aspects of model selection for the cerebrovascular diseases (CeVD) mortality data 11](#__RefHeading___Toc493965442)

[Table S2 13](#__RefHeading___Toc493965443)

[Table S3 14](#__RefHeading___Toc493965444)

[Table S4 15](#__RefHeading___Toc493965445)

[Derivation of correction factor to obtain city-averaged *EAR*-values for Table S4 16](#__RefHeading___Toc493965446)

[Aspects of model selection for the heart diseases mortality data 18](#__RefHeading___Toc493965447)

[Table S5 21](#__RefHeading___Toc493965448)

[Table S6 22](#__RefHeading___Toc493965449)

[Table S7 23](#__RefHeading___Toc493965450)

[Table S8 26](#__RefHeading___Toc493965451)

[References 28](#__RefHeading___Toc493965452)

Table S1 Number of individuals and deaths from CeVD and heart diseases (Shimizu et al. 2010)

| **Characteristics** | **Number of individuals**  **(*n* = 86611)** | **Number of deaths from CeVD (*n* = 9622)** | **Number of deaths from heart diseases**  **(*n* = 8463)** |
| --- | --- | --- | --- |
| Sex: |  |  |  |
| Male | 35687 | 3958 | 3261 |
| Female | 50924 | 5664 | 5202 |
| Age at atomic bomb exposure (years): |  |  |  |
| 0-9 | 17833 | 176 | 238 |
| 10-19 | 17563 | 404 | 508 |
| 20-29 | 10891 | 652 | 831 |
| 30-49 | 25774 | 4735 | 4575 |
| ≥50 | 14550 | 3655 | 2311 |
| Weighted colon dose (Gy): |  |  |  |
| <0.005 | 38509 | 4247 | 3723 |
| 0.005- | 23427 | 2637 | 2205 |
| 0.05- | 12508 | 1405 | 1305 |
| 0.2- | 6356 | 735 | 680 |
| 0.5- | 3424 | 363 | 342 |
| 1- | 1763 | 176 | 158 |
| ≥2 | 624 | 59 | 50 |

The table provides the number of participants and death cases from CeVD and heart diseases stratified by sex, age at exposure and weighted colon dose. All values were taken from Table 1 in Shimizu et al. (2010).

# Preston baseline model

The Preston baseline model has been developed for the study by Preston et al. (2003). It is as follows:

*h*_0_ = exp{*cm* + *cf* + *cmN* + *cfN* + *e30l70* × e30 × lage70 + *e30m* × e30 + *e30f* × e30

+ *e30sqm* × e30sq + *e30sqf* × e30sq + *e30qspm* × e30qsp + *e30qspf* × e30qsp

+ *e50qspm* × e50qsp + *e50qspf* × e50qsp + *l70m* × lage70 + *l70f* × lage70

+ *l70sqm* × lage70sq + *l70sqf* × lage70sq + *l40qspm* × lage40qsp + *l40qspf* × lage40qsp +

+ *l70qspm* × lage70qsp + *l70qspf* × lage70qsp} (S1)

with e30 := (*e* − 30)/10, e30sq := e30 × e30

e50 := (*e* − 50)/10

lage70 := ln(*a*/70), lage70sq := ln(*a*/70) × ln(*a*/70)

e30qsp := $\left\{\begin{matrix} 0 e\leq30 \\ \left[e30\right]\times\left[e30\right] e>30 \end{matrix}\right\}$

e50qsp := $\left\{\begin{matrix} 0 e\leq50 \\ \left[e50\right]\times\left[e50\right] e>50 \end{matrix}\right\}$

lage40qsp := $\left\{\begin{matrix} 0 a<40 \\ \ln^{2}\left(\frac{a}{40}\right) a\geq40 \end{matrix}\right\}$

lage70qsp := $\left\{\begin{matrix} 0 a<70 \\ \ln^{2}\left(\frac{a}{70}\right) a\geq70 \end{matrix}\right\}$

These naming conventions were taken from R13models.log available at <http://www.rerf.or.jp/library/dl_e/lss13.html>. Age at exposure is denoted by *e*, attained age by *a*. Model parameters in eq. (S1) are italicised. Parameters *cm*, *cf*, *cmN*, and *cfN* represent constant factors (*cm* and *cf*, for example, are constants related to males and females from Hiroshima), parameter *e30l70* describes variations of the hazard with multiplicative effects of attained age and age at exposure, while parameters *e30m* and *e30f* describe variations of the hazard with age at exposure for males and females, respectively. Parameters *e30qspm* and *e30qspf* mark the dependence on a quadratic spline function with age knot at *e* = 30 years for males and females, respectively. The Preston baseline model therefore uses 29 model parameters including the four age knots in e30qsp, e50qsp, lage40qsp, and lage70qsp.

# Mathematical functions used for the 13 parametric dose-response models from Figure 1

The general form of an ERR model is *h* = *h*_0_ × (1 + *ERR*(*D*, *s*, *a* , *e*)) where *h* is the total hazard function, *h*_0_ is the baseline model and the function *ERR*(*D*, *s*, *a* , *e*) describes the change of the hazard function with weighted colon dose *D* allowing for effects of sex (*s*), age at exposure (*e*) and attained age (*a*). It is *ERR*(*D*, *s*, *a* , *e*) = *err*(*D*) × ε(*s*, *a*, *e*) = *err*(*D*) × exp(*dem*_1_ × sex + *dem*_2_ × e30 + *dem*_3_ × lage70). Here, *err*(*D*) describes the shape of the dose-response function, ε(*s*, *a*, *e*) contains the dose-effect modifiers sex, attained age and age at exposure and *dem*_1_, *dem*_2_, and *dem*_3_ are three adjustable parameters related to the three dose-effect modifiers. The naming conventions for e30 and lage70 are provided after eq. (S1). The general form of an EAR model is *h* = *h*_0_ + *EAR*(*D*, *s*, *a* , *e*) where *EAR*(*D*, *s*, *a* , *e*) = *ear*(*D*) × ε(*s*, *a*, *e*). For *h*_0_ we first applied the Preston baseline model given in eq. (S1).

For *err*(*D*) the following dose-response models were used (analogous functions were applied for *ear*(*D*)).

*err*(*D*) = β_1_ × *D* LNT model

*err*(*D*) = 1.12 × β_1_ × *D*^2^ Quadratic (Q) model

*err*(*D*) = β_1_ × *D +* 1.12 × β_2_ × *D*^2^ Linear-quadratic (LQ) model

*err*(*D*) = β_1_ × *D* × exp(-β_2_ *D*) Linear-exponential (LE) model

$err(D)=\left\{\begin{matrix} 0 D<D_{th} \\ \beta_{1} \times(D-D_{th}) D\geq D_{th} \end{matrix}\right\}$ Linear-threshold (LTH) model

*err*(*D*) = 0.5 × *scale* × [tanh(10^5^ (*D* − *D_th_*)) – tanh(-10^5^ *D_th_*)] Step model

*err*(*D*) = 0.5 × *scale* × [tanh(*s* (*D* − *D_th_*)) – tanh(-*s* *D_th_*)] Smooth step model

$err(D)=\left\{\begin{matrix} 0.5\times scale\times[tanh(s(D-D_{th}))\text{ }-\text{ }tanh\left(-s\times D_{th}\right)] D<D_{th} \\ 0.5\times scale\times[tanh(s(D-D_{th}))\text{ }-\text{ }tanh\left(-s\times D_{th}\right)] + \beta_{1}\times(D-D_{th}) D\geq D_{th} \end{matrix}\right\}$ Step-linear model

$err(D)=\lambda_{0}\left(1-\frac{1}{1+\left(\frac{D}{D_{th}}\right)^{\lambda_{1}}}\right)$ Sigmoid model

*err*(*D*) = 1.12 × β_1_ × *D*×(*D-D*_th_) Hockey stick model (J-shaped model)

$err(D)=\lambda_{0}-\frac{\lambda_{0}+\lambda_{2}D}{1+\left(\frac{D}{D_{th}}\right)^{\lambda_{1}}}$ Hormesis model

$err(D)=\left\{\begin{matrix} \beta_{1}\times D D<D_{th} \\ \beta_{1} \times D_{th} + \beta_{2}\times(D-D_{th}) D\geq D_{th} \end{matrix}\right\}$ Two-line spline model

$err(D)=\left\{\begin{matrix} 0 D<0.005 \\ \beta_{1} 0.005\leq D<D_{1} \\ \beta_{2} D_{1} \leq D<D_{2} \\ \beta_{3} D_{2} \leq D<D_{3} \\ \beta_{4} D\geq D_{3} \end{matrix}\right\}$ Categorical model

The necessary adjustments for random errors in dosimetry applied to the dose term are already applied in the publicly available LSS data, but a separate adjustment involving a multiplication factor to the dose-squared covariable should be done explicitly, either according to Pierce et al. (1990) (factor 1.12) or Pierce et al. (2008) (revised factor 1.15). Since most of the published analyses apply the factor 1.12, this has been adopted here for the quadratic and linear-quadratic models and for the hockey stick model, which is nested with the quadratic model.

The step model generally gives a good indication where a threshold may be located. An explanation why this model generally yields a lower final deviance than a smooth step model is provided in the Appendix (section A.3) of Simonetto et al. (2014). Instantaneous changes in risk are, however, biologically unrealistic. The hormesis model was introduced by Brain and Cousens (1989).

# Figure S1

| 1 | 2 | 3 | 4 |
| --- | --- | --- | --- |
|  |  | sigmoid model | hormesis model |
|  |  |  |  |
|  |  | smooth step model | step-linear model |
|  |  |  |  |
|  | LTH model | two-line spline model |  |
| LNT model | LE model |  |  |
|  |  |  |  |
| Q model | LQ model |  |  |
|  |  |  |  |
|  | hockey stick model |  |  |

Number of model parameters for the applied dose-response models and relation between the models. Two models are nested if they are connected by an arrow.

# Calculation of AIC-weights

For a set of *n* non-nested models, the AIC-weight, *p_m_*, was calculated for model *m* according to the following equation (Burnham and Anderson 2002, Claeskens and Hjort 2008):

(S2)

Here, ΔAIC*_m_* = AIC*_m_* − AIC_0_, where AIC*_m_* is the AIC-value for model *m* and AIC_0_ is the smallest AIC-value of all *n* models. The resulting weights, multiplied by a factor of 10^4^, give the number of samples for risk estimates to be generated by uncertainty distribution simulations.

The criterion for inclusion of a model into the set of final non-nested models, which was used for multi-model inference (MMI), is whether p_1_ > 0.05 when comparing with the best model, i.e. the one with ΔAIC = 0 (Hoeting et al. 1999, Walsh 2007). In that case eq. (S2) reduces to *p*_m_ = exp(-ΔAIC_m_/2)/[exp(-ΔAIC_m_/2) + 1]. With this equation it is easy to show that for ΔAIC_1_ < 5.9 one obtains p_1_ > 0.05. Applying this formula to the final non-nested models for heart diseases (Table 1, main text), one finds for the ERR-smooth step and EAR-smooth step models *p*_1_ = 0.071 and 0.13, respectively. Consequently, these two models survive the selection process. The AIC-weights provided in Table 1 were calculated using eq. (S2) because to be useful for MMI they need to sum up to 1.

# Aspects of model selection for the cerebrovascular diseases (CeVD) mortality data

Table S2 provides the main results of fitting the parametric dose-response models from Figure 1 as ERR models to the mortality data for CeVD. Considering Figure S1 and applying the likelihood ratio test (LRT) three ERR models were identified for Occam’s group and were used for MMI. These three models are marked in bold in Table S2. The selection process was performed as follows. The final deviance (dev) of the ERR-LNT model was compared with the final deviance of the streamlined baseline model (dev = 13422.27). The improvement in deviance was Δdev = 4.74 > 3.81. Based on the LRT, the additional model parameter (β_1_) was therefore significant at the 95% significance level and the model was included into the set of final non-nested models that was used for MMI. For the ERR-Q model one obtains Δdev = 6.51 > 3.81 and so this model was also included for MMI. The LQ is nested with the LNT model and we obtained Δdev = 1.82 < 3.81. The additional parameter (β_2_) was therefore not significant, thereby eliminating the ERR-LQ model. For the ERR-LE model one finds Δdev = 1.5 < 3.81. The LTH model is nested with the LNT model. Comparing the final deviances we obtained Δdev = 2.19 < 3.81, which eliminated the ERR-LTH model. Because the step models (which were implemented as a modified hyperbolic tangent function) are not nested with any of the models that contain only one or two parameters (Figure S1), their deviances need to be compared with the one from the streamlined baseline model. The step model gives an indication of where a dose threshold may be located; for that model *D_th_* = 0.82 Gy was found. As explained in the previous section, slope *s* = 10^5^ Gy^-1^ was used and therefore this parameter does not count as a model parameter because 1/s ≈ 0 compared to all other parameter values. Compared to the streamlined baseline model this model therefore has two additional parameters and one finds Δdev = 13422.27 - 13415.48 = 6.79. Based on the LRT the two parameters *D_th_* and *scale* are therefore significant at the 95% level (6.79 > 5.99). This model is, however, biologically unrealistic because of its extremely steep slope. It was therefore not included for MMI. For the ERR-smooth step model with free slope parameter Δdev = 6.50 was found and because 6.50 < 7.82 this model was not included for MMI. The step-linear model is nested with the smooth step model but because the latter was not significant one compares with the streamlined baseline model to obtain Δdev = 6.70 < 9.49, which eliminated the ERR-step-linear model. The deviance of the sigmoid model, not nested with any of the models that contain only one or two parameters, was compared with the deviance of the streamlined baseline model to find Δdev = 6.67 < 7.81. Therefore, the sigmoid model did not survive the selection process as its three parameters were not significant. Although the hormesis model is nested with the sigmoid model, its deviance needs to be compared with the deviance of the streamlined baseline model because the sigmoid model was not significant. One obtains Δdev = 7.46 < 9.49. That eliminated the hormesis model. The hockey stick model is nested with the Q model but the improvement in quality of fit was marginal: Δdev = 0.06 < 3.84, i.e. the additional parameter *D_th_* was not significant. The two-line spline model is nested with the LTH model, which was, however, not significant. We therefore compared with the streamlined baseline model to obtain Δdev = 8.12 > 7.81. Consequently, the ERR-two-line spline model was included into the set of final non-nested models used for MMI. The problematic of applying the LRT in segmented regression (Feder 1975), as it is the case here for the two-line spline model, is known to the authors. One would have to simulate the deviance-distributions of the two models (ERR-two-line spline and streamlined baseline models); if the two distributions do not overlap by more than 5%, then they are significantly different and consequently β_1_, β_2_ and *D_th_* would be significant at the 95% level.

As described in the main text (*Methods* section), when the full Preston baseline model was streamlined in combination with an EAR-LNT model, a slightly different baseline model was obtained. The fit of this streamlined baseline model alone with its 17 parameters led to dev = 13417.91. The EAR-LNT model led to an improvement of Δdev = 3.70 < 3.84. For the EAR-Q model the improvement was not significant either: Δdev = 3.16 < 3.84. Both models were therefore not included for MMI. For the EAR-LQ, which is to be compared with the streamlined baseline model, the authors obtained Δdev = 3.72 < 5.99 and therefore its two parameters were not significant. Analogous results were obtained for the EAR-LTH model (Δdev = 4.08 < 5.99). The parameters of the EAR-smooth step and EAR-hockey stick models were not significant either. The EAR-sigmoid model yielded dev = 13414.02. The corresponding improvement compared to the streamlined baseline model was Δdev = 3.88 < 7.81. The EAR-hormesis model and the EAR-two-line spline model did not lead to stable fits.

Table S2 Results of fitting the dose-response models from Figure 1 as ERR models to the mortality data for CeVD (Shimizu et al. 2010)

|  | dev | Δdev | *N_par_* | AIC | ΔAIC |
| --- | --- | --- | --- | --- | --- |
| **ERR-LNT model** | **13417.53** | **3.38** | **16** | **13449.53** | **1.76** |
| **ERR-Q model** | **13415.77** | **1.61** | **16** | **13447.77** | **0** |
| ERR-LQ model | 13415.71 | 1.56 | 17 | 13449.71 | 1.94 |
| ERR-LE model | 13416.03 | 1.87 | 17 | 13450.03 | 2.26 |
| ERR-LTH model, *D_th_* = 0.48 Gy | 13415.34 | 1.18 | 17 | 13449.34 | 1.57 |
| ERR-step model, *D_th_* = 0.82 Gy | 13415.48 | 1.33 | 17 | 13449.48 | 1.72 |
| ERR-smooth step model, *D_th_* = 1.18 Gy | 13415.77 | 1.62 | 18 | 13451.77 | 4.01 |
| ERR-step-linear model, *D_th_* = 1.02 Gy | 13415.57 | 1.42 | 19 | 13453.57 | 5.81 |
| ERR-sigmoid model, *D_th_* = 1.34 Gy | 13415.60 | 1.45 | 18 | 13451.60 | 3.83 |
| ERR-hockey stick model, *D_th_* = 0.30 Gy | 13415.71 | 1.56 | 17 | 13449.71 | 1.94 |
| ERR-hormesis model, *D_th_* = 1.07 Gy | 13414.82 | 0.66 | 19 | 13452.82 | 5.05 |
| **ERR-two-line spline model**, *D_th_* = 0.14 Gy | **13414.15** | **0** | **18** | **13450.15** | **2.39** |

The models were implemented as ERR models. For each model resp. model fit the following measures are provided: the final deviance dev, Δdev, the number of model parameters *N_par_*, the Akaike Information Criterion (AIC) and ΔAIC. Here, the difference in final deviance is denoted by Δdev with respect to the model with the smallest final deviance. It is AIC = dev + 2 × *N_par_*. The difference in AIC-values with respect to the model with the smallest AIC-values is denoted by ΔAIC. Those models that were used for MMI are marked in bold. As a comparison, the fit of the streamlined baseline model alone with its 15 parameters led to dev = 13422.27.

Table S3 Model parameters, maximum likelihood estimates (MLEs) and Wald-type standard errors (in parenthesis) for the three final non-nested models that were used for MMI for the CeVD mortality data. Parameters 1 to 15 are the baseline parameters, parameters 16 to 18 are the radiation-associated parameters

| # | Parameter | ERR-LNT model | ERR-quadratic model | ERR-two-line spline model |
| --- | --- | --- | --- | --- |
| 1 | *cm* | -5.416 (0.026) | -5.412 (0.025) | -5.40 (0.027) |
| 1 | *cf* | -5.416 | -5.412 | -5.40 |
| 2 | *cmN* | 0.167 (0.034) | 0.165 (0.034) | 0.160 (0.034) |
| 3 | *cfN* | 0.058 (0.029) | 0.056 (0.029) | 0.050 (0.030) |
| 4 | *e30l70* (yr^-1^) | 0.367 (0.050) | 0.367 (0.050) | 0.369 (0.050) |
| 5 | *e30m* (yr^-1^) | 0.488 (0.017) | 0.488 (0.017) | 0.489 (0.017) |
| 6 | *e30f* (yr^-1^) | 1.476 (0.037) | 1.475 (0.037) | 1.462 (0.037) |
| 7 | *e30sqf* (yr^-2^) | 0.325 (0.015) | 0.32 (0.015) | 0.318 (0.014) |
| 8 | *e30qspf* (yr^-2^) | -0.380 (0.013) | -0.379 (0.013) | -0.372 (0.013) |
| 9 | *e50qspm* (yr^-2^) | -0.243 (0.035) | -0.243 (0.035) | -0.243 (0.035) |
| 10 | *e50qspf* (yr^-2^) | -0.140 (0.028) | -0.140 (0.028) | -0.141 (0.028) |
| 11 | *l70m* | 4.48 (0.11) | 4.48 (0.11) | 4.47 (0.11) |
| 12 | *l70f* | 5.32 (0.10) | 5.32 (0.10) | 5.32 (0.10) |
| 13 | *l70sqf* | 0.92 (0.26) | 0.92 (0.26) | 0.92 (0.26) |
| 14 | *e30agef*^a^ (yr) | 19.02 (0.40) | 19.02 (0.40) | 18.94 (0.40) |
| 15 | *e50agem*^b^ (yr) | 49.21 (1.51) | 49.19 (1.52) | 49.16 (1.52) |
| 15 | *e50agef* (yr) | 49.21 | 49.19 | 49.16 |
| 16 |  | β_1_ = 0.082 Gy^-1^ (0.039) | β_1_ = 0.051 Gy^-2^ (0.022) | β_1_ =-0.33 (0.23) |
| 17 |  |  |  | β_2_ *=*0.142 (0.052) |
| 18 |  |  |  | *D*_th_ *=*0.139 (0.022) |
|  |  |  |  |  |
|  | Final deviance | 13417.53 | 13415.77 | 13414.15 |

Parameter *cf* is linked to *cm*; parameter *e50agef* is linked to *e50agem*. The parameters *cf* and *e50agef* are therefore not counted to the number of model parameters (left column). ^a^ Denotes the age at exposure knot for females in e30qsp. ^b^ Denotes the age at exposure knot for males in e50qsp.

Table S4 Values for *ERR* and *EAR* for cerebrovascular diseases at 0.1 and 1 Gy calculated with MMI and with the three final non-nested models that were used for MMI. The risk values are provided for different values of age at exposure (*e*) and attained age (*a*)

| **CeVD** |  |  |
| --- | --- | --- |
|  | *ERR* | *EAR* (10^-4^ person-years) |
| Multi-model inference |  |  |
| 0.1 Gy |  |  |
| *e* = 20, *a* = 50 | -0.004 (-0.057, 0.013) | -0.025 (-0.40, 0.093) |
| *e* = 20, *a* = 70 | -0.004 (-0.057, 0.013) | -0.10 (-1.6, 0.37) |
| *e* = 30, *a* = 70 | -0.004 (-0.057, 0.013) | -0.16 (-2.6, 0.60) |
| *e* = 50, *a* = 70 | -0.004 (-0.057, 0.013) | -0.4 (-6.9, 1.6) |
| 1 Gy |  |  |
| *e* = 20, *a* = 50 | 0.0669 (0.0074, 0.14) | 0.460 (0.052, 0.97) |
| *e* = 20, *a* = 70 | 0.0669 (0.0074, 0.14) | 1.83 (0.20, 3.9) |
| *e* = 30, *a* = 70 | 0.0669 (0.0074, 0.14) | 2.99 (0.33, 6.3) |
| *e* = 50, *a* = 70 | 0.0669 (0.0074, 0.14) | 7.92 (0.87, 17) |
|  |  |  |
| Single models |  |  |
| 0.1 Gy, *e* = 30, *a* = 70 |  |  |
| ERR-LNT model | 0.00822 (5.2×10^-4^, 0.016) | 0.365 (0.023, 0.70) |
| ERR-Q model | 0.000576 (9.9×10^-5^, 0.0011) | 0.0257 (0.0045, 0.047) |
| ERR-two-line spline model | -0.033 (-0.076, 0.012) | -1.49 (-3.5, 0.51) |
| 1 Gy, *e* = 30, *a* = 70 |  |  |
| ERR-LNT model | 0.0822 (0.0052, 0.16) | 3.65 (0.23, 7.0) |
| ERR-Q model | 0.0576 (0.0099, 0.11) | 2.57 (0.45, 4.7) |
| ERR-two-line spline model | 0.0767 (-0.0012, 0.16) | 3.453 (-0.057, 7.1) |
|  |  |  |
| ERR-LNT model (Shimizu et al. 2010) | 0.09 (0.01, 0.17) | 2.3 (0.4, 4.4) |

The 95% confidence intervals are provided in parenthesis. The risk values from Shimizu et al. (2010) are also shown. The *ERR*-values are valid for males and females from both cities.

The general form of an ERR model is *h* = *h*_0_ × *RR* = *h*_0_ × (1 + *ERR*), where *RR* is the relative risk. Consequently, when we calculate the *EAR* from an ERR model we get *EAR* = *h* – *h*_0_ = *h*_0_ × (1 + *ERR*) – *h*_0_ = *h*_0_ × *ERR*. For CeVD, the baseline model, *h*_0_, depends on sex and city via model parameters *cm*, *cf*, *cmN*, and *cfN* (Table S3). Therefore, the *EAR*-values for CeVD in Table S4 also depend on sex and city: they are only valid for males from Hiroshima. The city-averaged *EAR*-values for men can be calculated by multiplication with a factor of 1.1 (the derivation of this factor is given in the following section). The *EAR*-values for women can be calculated by multiplying with a factor of 0.63.

When the *ERR* is calculated from an ERR model, then only the shape of the dose-responses related to the excess risk from radiation enters the risk estimate. Figures 2 and 3 in the main text are therefore valid for males and females from both cities.

# Derivation of correction factor to obtain city-averaged *EAR*-values for Table S4

In the previous section it has been shown that the *EAR* can be calculated from an ERR model as follows: *EAR* = *h*_0_ × *ERR*. The city-averaged *EAR*-values for males, <*EAR*>*_m_*, can be calculated as follows:

<*EAR*>*_m_* = *w_H_* × *EAR_H_* + *w_N_* × *EAR_N_* = *ERR* × (*w_H_* *h*_0_,*_mH_* + *w_N_* *h*_0_,*_mN_*) = *ERR* × (S3)

Here, *w_H_* = PY*_H_*/PY*_tot_* is the number of person years in Hiroshima divided by the total number of person years in the data set; *w_N_* = PY*_N_*/PY*_tot_* is the number of person years in Nagasaki divided by the total number of person years in the data set, and *h*_0_,*_mH_* is the streamlined baseline hazard for males in Hiroshima (with *cf* = *cmN* = *cfN* = 0; see eq. (S1)). An analogous definition holds for *h*_0_,*_mN_*, the streamlined baseline hazard for males in Nagasaki (with *cf* = *cfN* = 0).

Therefore, for the ERR-LNT model one finds

*h*_0_,*_mH_* = exp{*cm* + ….} = exp{−5.416} × exp{*rest*} where *rest* stands for all other terms in the exponential function.

It is

<*EAR*>*_m_* = *ERR* × *ERR* × $h_{0,mH}\times\left(w_{H}+w_{N}\frac{e^{cm+cmN}}{e^{cm}}\right)=$ *ERR* × $h_{0,mH}\times\left(w_{H}+w_{N}e^{cmN}\right).$ (S4)

Using *EAR* = *h*_0_ × *ERR* within Eq. (S4), one obtains

<*EAR*>*_m_* = *EAR_mH_* $\times\left(w_{H}+w_{N}e^{cmN}\right)$. (S5)

*EAR_mH_* stands for the *EAR*-values for males in Hiroshima as given in Table S4. The city-averaged *EAR*-values for males can be obtained by multiplication with the correction factor $\left(w_{H}+w_{N}e^{cmN}\right)$. It is *w_H_* = PY*_H_*/PY*_tot_* = 2197173.1/3294282.3 = 0.67 and *w_N_* = PY*_N_*/PY*_tot_* = 1097109.2/3294282.3 ≈ 0.33. Using the MLEs for *cmN* from Table S3, one finds that for all three final non-nested models the correction factor is approximately equal to 1.1. Consequently, the correction factor for the MMI-based *EAR*-values is also equal to 1.1. This factor can be applied to calculate from the *EAR*-values for MMI in Table S4 the city-averaged *EAR*-values (for example: for the MMI the *EAR*-value at 1 Gy for males in Hiroshima with an age attained of 70 years, exposed at an age of 30 years, is 2.99 per 10^4^ person-years. Therefore, the city-averaged *EAR*-value for males is 2.99 × 1.1 per 10^4^ person-years = 3.3 per 10^4^ person-years. This risk prediction holds for males in Hiroshima and Nagasaki).

# Aspects of model selection for the heart diseases mortality data

Table S5 provides the main results of fitting the parametric dose-response models from Figure 1 as ERR models to the mortality data for heart diseases. Considering Figure S1 and applying the LRT three non-nested ERR models were identified for Occam’s group and used for MMI. These three ERR models are marked in bold in Table S5. The selection process was performed as follows. The final deviance of the ERR-LNT model was compared with the deviance of the streamlined baseline model (dev = 13163.17). The improvement in deviance was Δdev = 10.65 > 3.81. Based on the LRT, the additional model parameter (β_1_) was therefore significant at the 95% significance level and the model was included into the set of final non-nested models that was used for MMI. For the ERR-Q model one obtains Δdev = 8.62 > 3.81 and so this model was also included for MMI. The LQ is nested with the LNT model and we obtained Δdev = 0.0035 < 3.81. The additional parameter β_2_ was therefore not significant, thereby eliminating the ERR-LQ model. For the ERR-LE model one finds Δdev = 0.0053 < 3.81 and it was therefore not included into Occam’s group. The LTH model is nested with the LNT model. Comparing the final deviances we obtained Δdev = 0.097 < 3.81, which eliminated the ERR-LTH model. The step model gives an indication of where a dose threshold may be located; for that model *D_th_* = 1.40 Gy was found. As for CeVD, slope *s* = 10^5^ Gy^-1^ was used and therefore this parameter does not count as a model parameter because 1/s ≈ 0 compared to all other parameter values. Compared to the streamlined baseline model this model therefore has two additional parameters and one finds Δdev = 13163.17 - 13153.23 = 9.94. Based on the LRT, the two parameters *D_th_* and *scale* were therefore significant at the 95% level (9.94 > 5.99). This model is, however, biologically unrealistic because of its extremely steep slope. It was therefore not included for MMI. For the ERR-smooth step model with free slope parameter one finds *s* = 118429 Gy^-1^ with Wald-type standard error 1.35×10^7^ Gy^-1^ and final deviance dev = 13153.08. Like before, a model with such an extremely steep slope is biologically unrealistic and was therefore not considered for MMI. Obviously, there is not enough information in the data to estimate the slope parameter with reasonable certainty. After some testing, it was decided to fix the slope parameter at a constant value of *s* = 10 Gy^-1^. This led to dev = 13153.66. Comparing this final deviance to the one of the streamlined baseline model one finds Δdev = 13163.17 – 13153.66 = 9.51 > 7.81 and therefore, this model was one of the final non-nested models to be used for MMI. The step-linear model is nested with the smooth step model and one finds Δdev = 0.14 < 3.81, which shows that the additional parameter (β_1_) was not significant at the 95% level. This eliminated the ERR-step-linear model. The deviance of the sigmoid model, not nested with any of the models that contain only one or two parameters, was compared with the deviance of the streamlined baseline model to find Δdev = 10.70 > 7.81. This model with its large MLEs for λ_0_ and *D_th_* is, however, essentially a linear fit with no threshold and was therefore not included for MMI because the LNT model was already included in the set of final non-nested models. The fit of the ERR-hormesis model, which is nested with the sigmoid model, led to Δdev = 2×10^-5^ < 3.81. That eliminated the hormesis model. The hockey stick model is nested with the Q model but the improvement in quality of fit shows that the additional parameter *D_th_* was not significant: Δdev = 2.03 < 3.84. The two-line spline model is nested with the LTH model, which was, however, not significant. We therefore compared with the streamlined baseline model to obtain Δdev = 13.45 > 7.81. Subsequently, sex turned out to be a significant dose-effect modifier. The resulting model did, however, not show any stability under cross-validation and was therefore not included for MMI.

As described in the main text (*Methods* section), when the full Preston baseline model was streamlined in combination with an EAR-LNT model, the same baseline model was obtained as the one found in combination with an ERR-LNT model. The EAR-LNT model led to an improvement of Δdev = 2.88 < 3.84 compared to the deviance of the streamlined baseline model (dev = 13163.17). Subsequently, the three dose-effect modifiers were tested for their significance and a significant effect modifier that depends on attained age was identified (Δdev = 9.07 > 3.84). Consequently, the two parameters β_1_ and *dem*_3_ (Table S7) together were significant: 11.94 > 5.99 (the problematic of applying the LRT when comparing a baseline model with a dose-response model that is multiplied with a dose-effect modifier (Liu and Shao 2003) is known to the authors). The EAR-LNT model was therefore included into the set of final non-nested models (i.e. Occam’s group) used for MMI (Tables S6 and S7). The EAR-Q model was significant: Δdev = 4.17 > 3.84. The age-dependent dose-effect modifier was also significant (7.22 > 3.84). This model was therefore included into Occam’s group and used for MMI. The EAR-LQ was to be compared with the EAR-Q model. Adding parameter *ear*_1_ leads to a marginal improvement of 0.08 deviance points. Consequently, this model was not included for MMI. The EAR-LE model needed to be compared with the streamlined baseline model because the EAR-LNT model was not significant. One finds Δdev = 8.79 > 5.99, i.e. parameters β_1_ and β_2_ (see section on mathematical functions of the dose-response models) together were significant. In addition, a significant attained age-dependent effect modifier was found. We therefore compared with the EAR-LNT model with age-dependent dose-effect modifier to find Δdev = 1.78 < 3.84. Consequently, the EAR-LE model was rejected. The EAR-LTH model is nested with the EAR-LNT model, which was, however, not significant. Comparing with the streamlined baseline model one finds Δdev = 10.87. No dose-effect modifier turned out to be significant. Consequently, the EAR-LTH model belongs to the set of final non-nested models (Tables S6 and S7). The biologically unrealistic step model yielded a final deviance dev = 13151.97. For the EAR-smooth step model with free slope parameter one finds *s* = 3413.71 Gy^-1^ with standard error 1.06×10^5^ Gy^-1^ and final deviance dev = 13152.02. Like before, a model with such an extremely steep slope is biologically unrealistic and was therefore not considered for MMI. As before with the ERR-smooth step model, there is not enough information in the data to determine the slope parameter with reasonable certainty and so *s* = 10 Gy^-1^ was applied. This led to dev = 13152.31. Comparing this final deviance to the one of the streamlined baseline model one finds Δdev = 13163.17 - 13152.31 = 10.85 > 7.81 and therefore, this model was one of the final non-nested models to be used for MMI. For the step-linear model one finds Δdev = 0.20, i.e. the additional parameter describing the linear slope for *D* ≥ *D_th_* was not significant at the 95% level. The deviance of the sigmoid model was compared with that of the streamlined baseline model: Δdev = 9.59 > 7.81. This model, however, increases dramatically at doses > 3 Gy. In that dose range only two cases of heart diseases can be found so that this steep dose-response is unsubstantiated. The fit of the EAR-hormesis model, nested with the EAR-sigmoid model, improves the quality of fit by only Δdev = 0.12 deviance points. That eliminated the hormesis model. Parameter *D_th_* of the hockey stick model was associated with Δdev = 0.079 compared to the EAR-Q model. Therefore, this model dropped out. The deviance of the two-line spline model was compared with the deviance of the EAR-LTH model yielding Δdev = 0.15. A significant age-dependent dose-effect modifier was found for the two-line spline model. Comparing with the EAR-LNT model with age-dependent dose-effect modifier yielded (Δdev = 13151.22 – 13146.02 = 5.2 < 5.99. That eliminated the two-Line spline model.

Table S5 Results of fitting the dose-response models from Figure 1 as ERR models to the mortality data for heart diseases (Shimizu et al. 2010)

|  | dev | Δdev | *N_par_* | AIC | ΔAIC |
| --- | --- | --- | --- | --- | --- |
| **ERR-LNT model** | **13152.52** | **6.73** | **21** | **13194.52** | **0.73** |
| **ERR-Q model** | **13154.54** | **8.75** | **21** | **13196.54** | **2.75** |
| ERR-LQ model | 13152.51 | 6.73 | 22 | 13196.51 | 2.73 |
| ERR-LE model | 13152.51 | 6.72 | 22 | 13196.51 | 2.72 |
| ERR-LTH model, *D_th_* = 0.01 Gy | 13152.42 | 6.63 | 22 | 13196.42 | 2.63 |
| ERR-step model , *D_th_* = 1.40 Gy | 13153.23 | 7.45 | 22 | 13197.23 | 3.45 |
| **ERR-smooth step model, *D_th_* = 1.52 Gy** | **13153.66** | **7.87** | **23** | **13199.66** | **5.87** |
| ERR-step-linear model, *D_th_* = 1.53 Gy | 13153.52 | 7.73 | 24 | 13201.52 | 7.73 |
| ERR-sigmoid model^1^, *D_th_* = 628.86 Gy | 13152.47 | 6.68 | 23 | 13198.47 | 4.68 |
| ERR-hockey stick model^1^, *D_th_* = -37.80 Gy | 13152.51 | 6.72 | 22 | 13196.51 | 2.72 |
| ERR-hormesis model^1^, *D_th_* = 631.85 Gy | 13152.47 | 6.68 | 24 | 13200.47 | 6.68 |
| ERR-two-line spline model, *D_th_* = 2.65 Gy | 13145.79 | 0 | 24 | 13193.79 | 0 |

The models were implemented as ERR models. For each model respectively model fit the following measures are provided: the final deviance dev, Δdev, the number of model parameters *N_par_*, the Akaike Information Criterion (AIC) and ΔAIC. Here, the difference in final deviance is denoted by Δdev with respect to the model with the smallest final deviance. It is AIC = dev + 2 × *N_par_*. The difference in AIC-values with respect to the model with the smallest AIC-values is denoted by ΔAIC. Those models that were used for MMI are marked in bold. As a comparison, the fit of the streamlined baseline model alone with its 20 parameters leds to dev = 13163.17.

^1^ The ERR-sigmoid, ERR-hockey stick and ERR-hormesis models lead to almost linear dose-responses.

Table S6 Results of fitting the dose-response models from Figure 1 as EAR models to the mortality data for heart diseases (Shimizu et al. 2010)

|  | dev | Δdev | *N_par_* | AIC | ΔAIC |
| --- | --- | --- | --- | --- | --- |
| **EAR-LNT model^a^** | **13151.22** | **5.20** | **22** | **13195.22** | **1.20** |
| **EAR-Q model^a^** | **13151.78** | **5.76** | **22** | **13195.78** | **1.76** |
| EAR-LQ model | 13150.90 | 4.87 | 23 | 13196.90 | 2.87 |
| EAR-LE model | 13149.44 | 3.42 | 23 | 13195.44 | 1.42 |
| **EAR-LTH model, *D_th_* = 2.36 Gy** | **13152.29** | **6.27** | **22** | **13196.29** | **2.27** |
| EAR-step model , *D_th_* = 2.54 Gy | 13151.97 | 5.95 | 22 | 13195.97 | 1.95 |
| **EAR-smooth step model, *D_th_* = 2.54 Gy** | **13152.31** | **6.29** | **23** | **13198.31** | **4.29** |
| EAR-step-linear model, *D_th_* = 2.50 Gy | 13152.11 | 6.09 | 24 | 13200.11 | 6.09 |
| EAR-sigmoid model, *D_th_* = 49.19 Gy | 13153.58 | 7.56 | 23 | 13199.58 | 5.56 |
| EAR-hockey stick model, *D_th_* = -2.89 Gy | 13150.90 | 4.88 | 23 | 13196.90 | 2.88 |
| EAR-hormesis model, *D_th_* = 49.60 Gy | 13153.46 | 7.44 | 24 | 13201.46 | 7.44 |
| EAR-two-line spline model, *D_th_* = 2.57 Gy | 13146.02 | 0.00 | 24 | 13194.02 | 0.00 |

The models were implemented as EAR models. As a comparison, the fit of the streamlined baseline model alone with its 20 parameters led to dev = 13163.17. See Table S5 for further explanations.

^a^ Contains an age-dependent dose-effect modifier

Table S7 Model parameters, MLEs and Wald-type standard errors (in parenthesis) for the seven final non-nested models that were used for MMI for the heart diseases mortality data

|  | Parameter | ERR-LNT | ERR-Q | ERR-smooth step | EAR-LNT |
| --- | --- | --- | --- | --- | --- |
| 1 | *cm* | -5.283 (0.032) | -5.27 (0.032) | -5.267 (0.031) | -5.275 (0.032) |
| 2 | *cf* | -8.04 (0.11) | -8.03 (0.12) | -8.02 (0.12) | -8.17 (0.12) |
| 3 | *cmN* | -0.025 (0.039) | -0.028 (0.039) | -0.027 (0.039) | -0.026 (0.040) |
| 4 | *cfN* | 0.082 (0.031) | 0.078 (0.031) | 0.077 (0.031) | 0.081 (0.031) |
| 5 | *e30l70* (yr^-1^) | 0.809 (0.074) | 0.809 (0.074) | 0.812 (0.074) | 0.819 (0.075) |
| 6 | *e30m* (yr^-1^) | 0.252 (0.018) | 0.252 (0.018) | 0.252 (0.018) | 0.254 (0.019) |
| 7 | *e30f* (yr^-1^) | 0.407 (0.021) | 0.407 (0.021) | 0.407 (0.021) | 0.419 (0.022) |
| 8 | *e30sqm* (yr^-2^) | -0.050 (0.010) | -0.050 (0.010) | -0.052 (0.010) | -0.053 (0.010) |
| 9 | *e30sqf* (yr^-2^) | -0.092 (0.011) | -0.092 (0.011) | -0.0928 (0.0098) | -0.0978 (0.0099) |
| 10 | *e50qspm* (yr^-2^) | -0.309 (0.079) | -0.309 (0.079) | -0.339 (0.093) | -0.341 (0.094) |
| 11 | *e50qspf* (yr^-2^) | -0.191 (0.051) | -0.191 (0.051) | -0.195 (0.047) | -0.199 (0.050) |
| 12 | *l70m* | 6.15 (0.14) | 6.15 (0.14) | 6.14 (0.14) | 6.14 (0.14) |
| 13 | *l70f* | 0.42 (0.33) | 0.42 (0.33) | 0.42 (0.33) | 0.13 (0.33) |
| 14 | *l70sqm* | 1.33 (0.19) | 1.33 (0.19) | 1.32 (0.19) | 1.32 (0.19) |
| 15 | *l70sqf* | -0.91 (0.26) | -0.91 (0.26) | -0.92 (0.26) | -1.03 (0.27) |
| 16 | *l40qspf* | 7.48 (0.31) | 7.48 (0.31) | 7.49 (0.32) | 7.72 (0.31) |
| 17 | *l70qspf* | -9.41 (1.00) | -9.41 (1.00) | -9.43 (1.00) | -9.7 (1.0) |
| 18 | *l40agef* | 40.99 (0.55) | 40.99 (0.55) | 40.99 (0.56) | 40.71 (0.53) |
| 19 | *l70agef*^a^ (yr) | 70 | 70 | 70 | 70 |
| 20 | *e50agem*^b^ (yr) | 54.6 (1.8) | 54.6 (1.8) | 55.5 (2.1) | 55.5 (2.1) |
| 20 | *e50agef* (yr) | 54.6 | 54.6 | 55.5 | 55.5 |
| 21 |  | β_1_ = 0.136 Gy^-1^ (0.044) | β_1_ = 0.064 Gy^-2^ (0.024) | *s* = 10 Gy^-1^ | β_1_ = 0.00055 Gy^-1^ pyr^-1^ (0.00020) |
| 22 |  |  |  | *scale* = 0.40 (0.15) | *dem*_3_ = 5.3 (2.2) |
| 23 |  |  |  | *D_th_* = 1.52 Gy (0.12) |  |
|  |  |  |  |  |  |
|  | Final deviance | 13152.52 | 13154.54 | 13153.66 | 13151.22 |

**Table S7 continued**

|  | Parameter | EAR-Q | EAR-LTH | EAR-smooth step |
| --- | --- | --- | --- | --- |
| 1 | *cm* | -5.27 (0.032) | -5.262 (0.031) | -5.262 (0.031) |
| 2 | *cf* | -8.17 (0.12) | -8.16 (0.12) | -8.17 (0.12) |
| 3 | *cmN* | -0.028 (0.040) | -0.028 (0.039) | -0.028 (0.039) |
| 4 | *cfN* | 0.077 (0.031) | 0.077 (0.031) | 0.077 (0.031) |
| 5 | *e30l70* (yr^-1^) | 0.813 (0.074) | 0.808 (0.074) | 0.808 (0.074) |
| 6 | *e30m* (yr^-1^) | 0.253 (0.019) | 0.252 (0.018) | 0.252 (0.018) |
| 7 | *e30f* (yr^-1^) | 0.413 (0.021) | 0.411 (0.021) | 0.411 (0.021) |
| 8 | *e30sqm* (yr^-2^) | -0.052 (0.010) | -0.052 (0.010) | -0.052 (0.010) |
| 9 | *e30sqf* (yr^-2^) | -0.0960 (0.0098) | -0.0954 (0.0097) | -0.0954 (0.0097) |
| 10 | *e50qspm* (yr^-2^) | -0.341 (0.094) | -0.340 (0.094) | -0.340 (0.094) |
| 11 | *e50qspf* (yr^-2^) | -0.201 (0.050) | -0.200 (0.050) | -0.201 (0.050) |
| 12 | *l70m* | 6.15 (0.14) | 6.14 (0.14) | 6.14 (0.14) |
| 13 | *l70f* | 0.14 (0.33) | 0.16 (0.33) | 0.15 (0.33) |
| 14 | *l70sqm* | 1.33 (0.19) | 1.33 (0.19) | 1.33 (0.19) |
| 15 | *l70sqf* | -1.02 (0.26) | -1.01 (0.26) | -1.01 (0.26) |
| 16 | *l40qspf* | 7.57 (0.30) | 7.45 (0.30) | 7.45 (0.30) |
| 17 | *l70qspf* | -9.50 (1.00) | -9.29 (0.99) | -9.28 (0.99) |
| 18 | *l40agef* | 40.40 (0.53) | 40.23 (0.53) | 40.21 (0.53) |
| 19 | *l70agef*^a^ (yr) | 70 | 70 | 70 |
| 20 | *e50agem*^b^ (yr) | 55.5 (2.1) | 55.5 (2.1) | 55.6 (2.1) |
| 20 | *e50agef* (yr) | 55.5 | 55.5 | 55.6 |
| 21 |  | β_1_ = 0.00022 Gy^-2^ pyr^-1^ (0.00011) | β_1_ = 0.0020 Gy^-1^ pyr^-1^ (0.0013) | *s* = 10 Gy^-1^ |
| 22 |  | *dem*_3_ = 4.00 (1.66) | *D_th_* = 2.36 Gy (0.16) | *scale* = 0.00091 pyr^-1^ (0.00051) |
| 23 |  |  |  | *D_th_* = 2.54 Gy (0.18) |
|  |  |  |  |  |
|  | Final deviance | 13151.78 | 13152.29 | 13152.31 |

Parameter *e50agef* is linked to *e50agem* and is therefore not counted to the number of model parameters (left column).

^a^ Denotes the attained age knot for females in lage70qsp. This parameter was treated as a constant.

^b^ Denotes the age at exposure knot for males in e50qsp.

Table S8 Values for *ERR* and *EAR* for heart diseases at 1 Gy calculated with MMI and with the seven final non-nested ERR and EAR models that were used for MMI. The risk values are provided for different values of age at exposure (*e*) and attained age (*a*)

| **Heart diseases** |  |  |
| --- | --- | --- |
|  | *ERR* | *EAR* (10^-4^ person-years) |
| Multi-model inference |  |  |
| 1 Gy |  |  |
| *e* = 20, *a* = 50 | 0.09 (0, 0.26) | 0.7 (0, 1.9) |
| *e* = 20, *a* = 70 | 0.09 (0, 0.22) | 3.5 (0, 8.4) |
| *e* = 30, *a* = 70 | 0.08 (0, 0.20) | 4 (0, 10) |
| *e* = 50, *a* = 70 | 0.07 (0, 0.20) | 5 (0, 13) |
|  |  |  |
| Single models |  |  |
| 1 Gy, *e* = 30, *a* = 70 |  |  |
| ERR-LNT model | 0.136 (0.050, 0.22) | 6.9 (2.5, 11) |
| ERR-Q model | 0.072 (0.019, 0.12) | 3.67 (0.99, 6.3) |
| ERR-smooth step model | 1.3×10^-5^ (< 10^-7^, 0.0013) | 6.6×10^-4^ (< 10^-5^, 0.067) |
| EAR-LNT model | 0.108 (0.029, 0.19) | 5.5 (1.5, 9.5) |
| EAR-Q model | 0.0488 (0.0028, 0.096) | 2.52 (0.15, 4.9) |
| EAR-LTH model | 0 | 0 |
| EAR-smooth step model | 0 | 0 |
|  |  |  |
| ERR-LNT model (Shimizu et al. 2010) | 0.14 (0.06, 0.23) | 3.2 (1.3, 5.2) |

The 95% confidence intervals are provided in parenthesis. The risk values from Shimizu et al. (2010) are also shown. The MMI-based *ERR*- and *EAR*-values are valid for males from Hiroshima. The *ERR*-values that are based on the three ERR models are valid for males and females of both cities, analogous for the *EAR*-values of the four EAR models.

When calculating the *EAR* from an ERR model one finds *EAR* = *h*_0_ × *ERR*. For heart diseases, *h*_0_ depends on sex and city via model parameters *cm*, *cf*, *cmN*, and *cfN* (Table S7). Therefore, the *EAR*-values for the three non-nested ERR models (ERR-LNT, ERR-Q and ERR-smooth step) in Table S8 also depend on sex and city: they are only valid for males from Hiroshima. The city-averaged *EAR*-values for men can be calculated by multiplication with the factor (*w_H_* + *w_N_* e*^cmN^*) that evaluates to 0.99 for each of the three ERR models (the derivation of this factor is analogous to the one shown below Table S4). The *EAR*-values for women can be calculated by multiplying with a factor of 0.54.

The general form of the EAR model is *h* = *h*_0_ + *EAR*. When the *ERR* is calculated from an EAR model, one gets *ERR* = *h*/*h*_0_ − 1 = (*h*_0_ + *EAR*)/*h*_0_ − 1 = *EAR*/*h*_0_. For heart diseases, the baseline model, *h*_0_, dependents on city and sex via model parameters *cm*, *cf*, *cmN*, and *cfN* (Table S7). Therefore, the *ERR*-values for the four non-nested EAR models in Table S8 also depend on city and sex: they are only valid for males from Hiroshima. The city-averaged *ERR*-values for men can be calculated by multiplication with the factor (*w_H_* + *w_N_* e*^-cmN^*) that evaluates to 1.01 for each of the four EAR models. The *ERR*-values for women can be calculated by multiplying with factors 1.86 and 1.85 for the EAR-LNT and EAR-Q models, respectively.

The above mentioned correction factors for females need to be applied to calculate the MMI-based *ERR*- and *EAR*-values for females. For example, the MMI-based ERR-value for females at 1 Gy for *e* = 30 years and *a* = 70 years is 0.11.

For Figure 4 in the main text the *ERR* has in part been calculated from EAR models. Figure 4 is therefore only valid for males from Hiroshima. For Figure 5 the *EAR* has in part been calculated from ERR models. Following the arguments given below Table S4, Figure 5 is therefore only valid for males from Hiroshima.

References

Brain P, Cousens R (1989) An equation to describe dose responses where there is stimulation of growth at low doses. Weed Research 29:93–96

Burnham KP, Anderson DR (2002) Model selection and multimodel inference. 2nd ed. New York: Springer

Claeskens G, Hjort NL (2008) Model selection and model averaging. Cambridge University Press, Cambridge

Feder PI (1975) The log likelihood ratio in segmented regression. The Annals of Statistics 3:84-97

Hoeting JA, Madigan D, Raftery AE, Volinsky CT (1999) Bayesian model averaging: a tutorial. Statist Sci 14:382–417

Liu BX, Shao Y (2003) Asymptotics for likelihood ratio tests under loss of identifiability. The Annals of Statistics 31: 807–832

Pierce DA, Stram DO, Vaeth M (1990) Allowing for random errors in radiation dose estimates for the atomic bomb survivor data. Radiat Res 123:275–284

Pierce DA, Vaeth M, Cologne J (2008) Allowance for random dose estimation errors in atomic bomb survivor studies: a revision. Radiat Res 170:118–126

Preston DL, Shimizu Y, Pierce DA, Suyama A, Mabuchi K (2003) Studies of mortality of atomic bomb survivors. Report 13: solid cancer and noncancer disease mortality: 1950–1997. Radiat Res 160:381–407

Shimizu Y, Kodama K, Nishi N, Kasagi F, Suyama A, Soda M, Grant EJ, Sugiyama H, Sakata R, Moriwaki H, Hayashi M, Konda M, Shore RE (2010) Radiation exposure and circulatory disease risk: Hiroshima and Nagasaki atomic bomb survivor data, 1950–2003. Brit Med J 340:b5349

Simonetto C, TV Azizova, ES Grigoryeva, JC Kaiser, H Schöllnberger, M Eidemüller (2014) Ischemic heart disease in workers at Mayak PA: Latency of incidence risk after radiation exposure. PLOS ONE 9:e96309

Walsh L (2007) A short review of model selection techniques for radiation epidemiology. Radiat Environ Biophys 46:205-213
